# Microbiota dynamics in a randomized trial of gut decontamination during allogeneic hematopoietic cell transplantation

**DOI:** 10.1172/jci.insight.154344

**Published:** 2022-04-08

**Authors:** Christopher J. Severyn, Benjamin A. Siranosian, Sandra Tian-Jiao Kong, Angel Moreno, Michelle M. Li, Nan Chen, Christine N. Duncan, Steven P. Margossian, Leslie E. Lehmann, Shan Sun, Tessa M. Andermann, Olga Birbrayer, Sophie Silverstein, Carol G. Reynolds, Soomin Kim, Niaz Banaei, Jerome Ritz, Anthony A. Fodor, Wendy B. London, Ami S. Bhatt, Jennifer S. Whangbo

**Affiliations:** 1 Department of Pediatrics, Division of Pediatric Hematology/Oncology and Division of Pediatric Stem Cell Transplant and Regenerative Medicine,; 2 Departments of Genetics and Medicine, Division of Hematology,; 3 Departments of Biology and Biomedical Informatics, and; 4 Department of Pathology, Stanford University, Palo Alto, California, USA.; 5 Department of Biomedical Informatics, Harvard Medical School, Boston, Massachusetts, USA.; 6 Dana-Farber/Boston Children’s Cancer and Blood Disorders Center, Boston, Massachusetts, USA.; 7 Harvard Medical School, Boston, Massachusetts, USA.; 8 Department of Bioinformatics and Genomics, College of Computing and Informatics, University of North Carolina at Charlotte, Charlotte, North Carolina, USA.; 9 Department of Medicine, Division of Infectious Diseases, University of North Carolina at Chapel Hill, Chapel Hill, North Carolina, USA.; 10 Dana-Farber Cancer Institute, Boston, Massachusetts, USA.; 11 Department of Medicine, Division of Infectious Diseases, Stanford University, Palo Alto, California, USA.

**Keywords:** Hematology, Infectious disease, Bacterial infections, Molecular genetics, Stem cell transplantation

## Abstract

**BACKGROUND:**

Gut decontamination (GD) can decrease the incidence and severity of acute graft-versus-host disease (aGVHD) in murine models of allogeneic hematopoietic cell transplantation (HCT). In this pilot study, we examined the impact of GD on gut microbiome composition and the incidence of aGVHD in HCT patients.

**METHODS:**

We randomized 20 patients undergoing allogeneic HCT to receive (GD) or not receive (no-GD) oral vancomycin-polymyxin B from day –5 through neutrophil engraftment. We evaluated shotgun metagenomic sequencing of serial stool samples to compare the composition and diversity of the gut microbiome between study arms. We assessed clinical outcomes in the 2 arms and performed strain-specific analyses of pathogens that caused bloodstream infections (BSI).

**RESULTS:**

The 2 arms did not differ in the predefined primary outcome of Shannon diversity of the gut microbiome at 2 weeks post-HCT (genus, *P* = 0.8; species, *P* = 0.44) or aGVHD incidence (*P* = 0.58). Immune reconstitution of T cell and B cell subsets was similar between groups. Five patients in the no-GD arm had 8 BSI episodes versus 1 episode in the GD arm (*P* = 0.09). The BSI-causing pathogens were traceable to the gut in 7 of 8 BSI episodes in the no-GD arm, including *Staphylococcus* species.

**CONCLUSION:**

While GD did not differentially affect Shannon diversity or clinical outcomes, our findings suggest that GD may protect against gut-derived BSI in HCT patients by decreasing the prevalence or abundance of gut pathogens.

**TRIAL REGISTRATION:**

ClinicalTrials.gov NCT02641236.

**FUNDING:**

NIH, Damon Runyon Cancer Research Foundation, V Foundation, Sloan Foundation, Emerson Collective, and Stanford Maternal & Child Health Research Institute.

## Graphical abstract

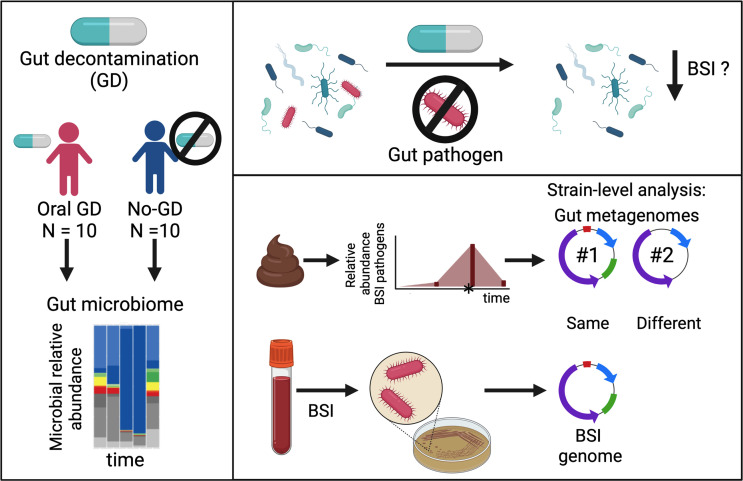

## Introduction

Despite advances in graft manipulation and acute graft-versus-host disease–prophylactic (aGVHD-prophylactic) regimens, treatment-related complications remain major causes of morbidity and mortality in patients undergoing allogeneic hematopoietic cell transplantation (allo-HCT). In efforts to decrease the incidence and severity of aGVHD, gut decontamination (GD) with nonabsorbable antibiotics in the peri-HCT time period is administered in some clinical centers, largely on the basis of results from limited preclinical studies. Specifically, mouse and human studies have demonstrated that nonabsorbable antibiotics can debulk intestinal bacteria and that this can decrease aGVHD (1–5). While the precise mechanism of how GD affects aGVHD remains unknown, it is thought that decreasing microbial load or altering the microbiota composition can improve intestinal barrier integrity and decrease inflammation via interactions with the host immune system (reviewed in refs. 6, 7).

In addition to nonabsorbable antibiotics, a distinct practice of systemic antimicrobial prophylaxis has also been used with the intent of suppressing bacterial growth in the gut to alter clinical outcomes. For example, Beelen et al. showed that patients randomized to receive ciprofloxacin plus metronidazole had a lower incidence of grade II–IV aGVHD compared with those randomized to receive ciprofloxacin alone (25% vs. 50%, *P* < 0.01) (8), suggesting a link between anaerobic bacteria and aGVHD. However, other studies have shown that the use of systemic antibiotics with activity against anaerobic bacteria is associated with an increased risk of aGVHD (9–11). If the effect of GD on the pathogenesis of aGVHD is related to alteration of the microbiota composition, then measuring the impact of GD on the microbiota may help resolve these discordant findings.

Prior clinical trials for GD are confounded by the variations in the practice of GD and systemic prophylaxis across centers with no consensus regarding the choice of antibiotic regimen (reviewed in ref. 7). In addition, prior studies relied on culture-based approaches to measure microbiome composition. However, many organisms within the gut microbiota, such as strict anaerobes, are difficult to culture (7). Modern approaches based on next-generation sequencing (NGS) enable more comprehensive profiling of the gut microbiome composition by overcoming the need to maintain viability of the organisms within the stool sample and thus may help inform the specific impact of GD on the gut microbiota.

While aGVHD is the most prevalent and fatal treatment-related complication of HCT, bloodstream infections (BSIs) are also an important cause of treatment-related morbidity and mortality in patients undergoing allo-HCT. The cumulative incidence of BSI in pediatric HCT patients is approximately 20% in the first 100 days (12) (ranging from 15% to 65%; refs. 12–16), with 18% BSI-attributable mortality (range, 12% to 20%) (12) and estimated health care cost of $40,000–$70,000 per BSI incident (13, 17). In adult HCT patients, a diverse gut microbiome is associated with a lower risk of chemotherapy-related BSI (16). Multiple studies have shown the microbiota composition may predict BSI in adult (16, 18) and pediatric HCT patients (19, 20) and in pediatric patients undergoing chemotherapy for acute leukemia (21). GD has been explored in the granulocytopenic population as a strategy to reduce BSI (22), in patients in intensive care units (ICUs) regardless of underlying diagnosis (23, 24), and to a limited extent in HCT patients, with no difference compared to historical controls (25). The ICU studies have reported conflicting results, with some studies showing a reduction in BSI (23), while others report no significant differences in Gram-negative bacteremia (24).

BSIs in HCT patients are the consequence of infectious pathogens entering the bloodstream through indwelling catheters, breakdown in the skin, and mucosal barrier injury (MBI) secondary to conditioning chemotherapy and resulting neutropenia. Changes in the abundance of MBI-associated enteric strains can precede BSI episodes in patients undergoing HCT (16, 19, 26). In addition to MBI, different bacterial strains of the same species can respond differently to selective pressures (27), with certain strains being more fit than other closely related ones (28, 29). Being able to identify and subsequently analyze strain variation (30) could help define how BSIs occur in patients.

We carried out what we believe is the first prospective, randomized study of GD using oral, nonabsorbable antibiotics (vancomycin and polymyxin B) to assess the impact of GD on the gut microbiome composition and diversity, with secondary outcomes including aGVHD and immune reconstitution. GD with vancomycin-polymyxin B in HCT patients was a long-standing institutional practice at Boston Children’s Hospital (BCH) due to these antibiotics’ broad Gram-positive and Gram-negative coverage and lack of systemic absorption. However, the effect of vancomycin-polymyxin B on the gut microbial diversity during allo-HCT is not known. While historical studies suggest a trend of GD decreasing GVHD, studies in the last decade (9–11, 31) have suggested that decreased diversity of the gut microbiota leads to worse clinical outcomes. Given this discrepancy, we sought to compare the institutional practice of GD against the practice of no-GD to determine if there is a difference in Shannon diversity and clinical outcomes in a single center. In an exploratory analysis, we investigated whether GD is associated with a decreased incidence of BSI.

## Results

### Patient characteristics are similar between the 2 study arms.

**Figure 1:**
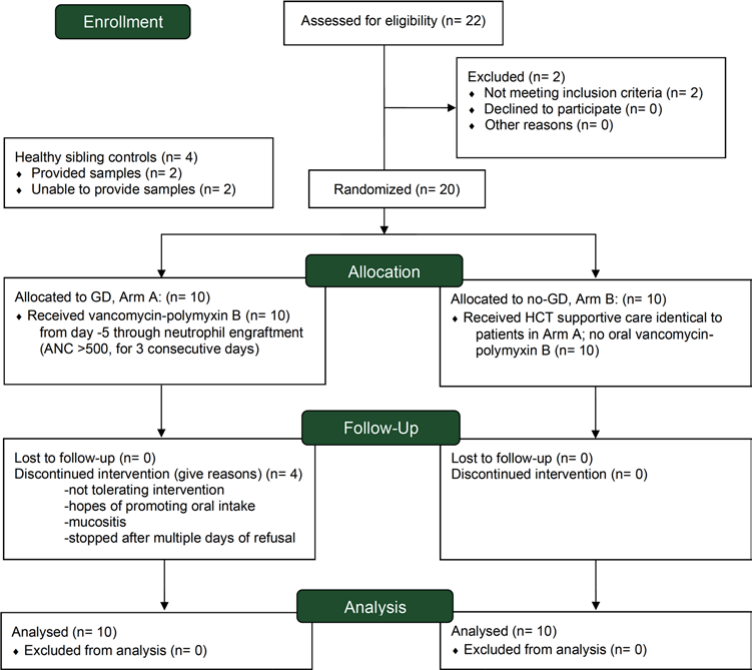
Study flow diagram. ClinicalTrials.gov Identifier NCT02641236. Self-reported racial and ethnic categories in Supplemental Table 11. ANC, absolute neutrophil count.

**Figure 2:**
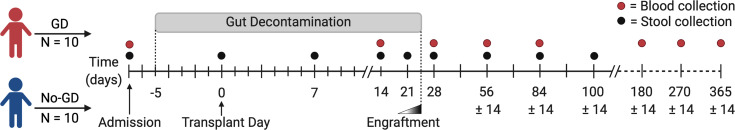
Study design. Twenty patients undergoing allo-HCT were randomized to 2 arms, 10 patients with GD and 10 patients with no GD. The GD arm received vancomycin-polymyxin B starting day –5 through engraftment (median neutrophil engraftment day +25, see Supplemental Figure 1) and was analyzed as intention-to-treat (Supplemental Table 1). The no-GD arm had the same stool and blood collection time points and did not receive oral vancomycin-polymyxin B. Black circles show time of stool collections, including pretransplant, weekly until engraftment, and monthly until day +100. An additional cohort of 2 healthy sibling donors serve as a stool control comparison group (Supplemental Figure 8). For immune reconstitution studies, blood samples (red circles) were collected at pretransplant, at 2 weeks, monthly for the first 3 months, and then at months 6, 9, and 12.

Twenty patients undergoing allogeneic HCT were enrolled and randomized between March 2016 and June 2019 (Figure 1). Ten patients received GD per BCH standard practice with oral nonabsorbable vancomycin-polymyxin B from day –5 relative to the transplant through neutrophil engraftment (dosing details in Supplemental Table 1; supplemental material available online with this article; https://doi.org/10.1172/jci.insight.154344DS1; actual administration in Supplemental Figure 1), and 10 patients received no-GD (Figure 2). Baseline characteristics in the 2 arms were similar (Table 1). The median age at HCT for all patients was 15.2 years (range, 7.10–24.6). Most patients had underlying hematologic malignancies (*n* = 15), received myeloablative conditioning regimens (*n* = 17), and had bone marrow as the graft source (*n* = 19). A median of 7 stools (range, 3–18) were collected per patient starting pretransplant through 1 year posttransplant. Of the stool samples, 76% were collected within the first 30 days after HCT (Supplemental Figure 2).

### Shannon diversity decreases similarly in patients with or without GD.

To compare gut microbiome Shannon diversity between individuals on the 2 arms of the study, we first determined the taxonomic composition of the gut microbiota using time-series stool collections. DNA was extracted from 147 patient stool samples, and after DNA library preparation, 142 (97%) samples were able to be sequenced using whole-genome shotgun (WGS) short-read sequencing (Supplemental Figure 2; 5 of 147, or 3%, had insufficient biomass to be sequenced). Libraries were sequenced to a median depth of 72.3 × 10^6^ read pairs (range, 3.7 × 10^6^ to 338 × 10^6^) per stool sample. After preprocessing and quality control filtering of reads, a median of 16.9 × 10^6^ (range, 4.3 × 10^4^ to 81 × 10^6^) high-quality reads per sample were obtained (Supplemental Figure 3). In most cases where a large proportion of reads was removed during preprocessing (*n* = 2 with fewer than 1 × 10^5^ reads; *n* = 14 of 142, or 10%, with fewer than 1 × 10^6^ reads), this was due to a very high proportion of human reads within the sample. Taxonomic composition was determined using Kraken2 classification (32) against a database of all bacterial, fungal, and viral genomes contained in National Center for Biotechnology Information’s (NCBI) GenBank as of January 2020.

**Figure 3:**
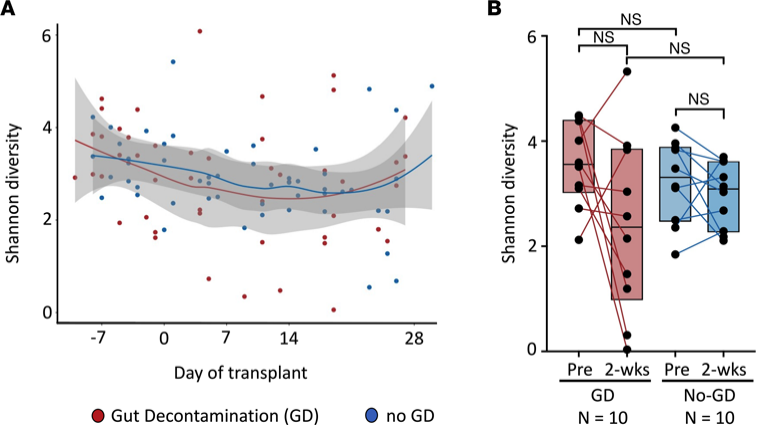
Shannon diversity is similar between the GD and no-GD groups based on intention-to-treat analysis at the species taxonomic level. Samples from patients undergoing GD (red) and no GD (blue). (**A**) Shannon diversity over time analyzed at the species level using local polynomial regression fitting (LOESS, locally estimated scatterplot smoothing of the mean Shannon diversity) showing similarity between the 2 groups. *n* = 48 samples from 10 patients in GD arm, *n* = 51 samples from 10 patients in no-GD arm. (**B**) Shannon diversity of individual patients from pretransplant (before GD antibiotics) to 2 weeks after HCT connected with a line. Boxes shown are the median with hinges at the 25% and 75%. All comparisons not significant (see Supplemental Table 4 for details) using Wilcoxon rank-sum test. *n* = 10 GD arm, *n* = 10 no-GD arm.

To determine if GD altered diversity of the gut microbiota, we focused on the primary endpoint of Shannon diversity at 2 weeks after HCT. At this time point, stool specimens likely reflect changes induced by the conditioning regimen and GD in the GD arm, are collected prior to the development of inflammation and aGVHD (33), and usually precede the use of immunosuppressive medications for treatment of aGVHD. Shannon diversity, which is a measurement sensitive to the loss of rare taxa (34) and estimates microbial richness (e.g., the number of species) and evenness (e.g., the relative abundance of organisms within a sample), was calculated for each sample (Supplemental Figure 4). Consistent with previous studies (16, 31, 35), the median Shannon diversity of the gut microbiota at the species level prior to GD exposure was 3.6 (range, 2.1–4.5) for GD and 3.3 (range, 1.2–4.2) for no-GD and decreased at 2 weeks posttransplant to 2.4 (range, 0.03–5.32) for GD and 3.1 (range, 2.1–3.7) for no-GD (Figure 3A, Supplemental Table 3, and Supplemental Figure 5A for genus). Shannon diversity in the 2 arms was similar at baseline prior to GD exposure (Figure 3B for species: *P* = 0.35; Supplemental Figure 5B for genus: *P* = 0.32; Supplemental Table 4). At 2 weeks posttransplant, there were no apparent differences in Shannon diversity between the GD and no-GD arms at the species (*P* = 0.44, Figure 3B) or genus level (*P* = 0.80, Supplemental Figure 5B), or change in Shannon diversity from baseline (Supplemental Figure 6). Furthermore, in an exploratory analysis, there was similar Shannon (α) diversity between the 2 arms when extending the window beyond 30 days to include all samples in the study (Supplemental Figure 7). Between the 2 arms, β diversity appeared similar (Supplemental Figure 8), with the exception of a group of outliers in samples from 3 patients with more than 45% relative abundance of *Enterococcus faecium* (*E*. *faecium*). As a control, stool samples from 2 healthy sibling donors were also collected to serve as a comparison to the HCT patients, which were similar to each other and the HCT siblings at the time points collected (based on analysis of similarity, ANOSIM, in Supplemental Figure 8 legend). Based on these findings, there is no evidence of a significant difference in Shannon diversity in our intention-to-treat analysis of the gut microbiota between the 2 arms.

### No difference in cumulative exposure to antibiotics between GD and no-GD arms.

We sought to interrogate why gut microbiome Shannon diversity did not differ between the 2 arms. While adherence to vancomycin-polymyxin B varied (Supplemental Figure 1), we found no correlation between the proportion of GD doses received and Shannon diversity within the GD arm (Supplemental Figure 9). Unfortunately, the small sample size precludes a robust analysis comparing the microbial communities between patients with good adherence (>70% of planned doses, *n* = 4) versus those who had poor adherence (<30% doses, *n* = 2).

As GD might affect pathogen colonization in the gut microbiota and possibly subsequent bloodstream translocation of these pathogens, we postulated that GD might be associated with decreased fevers and consequently decreased overall antibiotic exposure. In an exploratory analysis of the difference in systemic antibiotics between the 2 arms, we analyzed the clinical records based on individual antibiotics, class of antibiotics, and clinical indication including broad-spectrum antibiotics with anaerobic coverage (e.g., piperacillin-tazobactam, meropenem). We found that the duration of prophylactic and therapeutic antibiotic exposure (within 30 days after HCT) was similar between the 2 treatment arms (Supplemental Table 5), including no difference in exposure to broad-spectrum antibiotics with anaerobic coverage (median 13 days GD versus 17 days no-GD, *P* = 0.68). Thus, it is possible that the impact of GD on the microbiota was small compared with the impact of systemic broad-spectrum antibiotics.

### Comparison of secondary clinical outcomes.

The prespecified secondary outcomes of this study were stool frequency in the first 7 days, incidence of aGVHD in the first 100 days, relapse-free survival, overall survival (OS), and immune cell reconstitution. There was no apparent difference in the incidence of diarrhea in the first 7 days posttransplant between the 2 treatment arms (Table 2, *P* = 1.0). The overall incidence of grade II–IV aGVHD was 20%, with 1 patient in the GD arm and 3 patients in the no-GD arm (*P* = 0.58, Table 2). The median day of onset of aGVHD was 38 days post-HCT (range 24–63). Of the 15 patients with a malignancy, 3 patients in the GD arm (*n* = 7) and 2 patients in the no-GD arm (*n* = 8) had malignant relapse within 2 years after HCT; the 1-year relapse-free survival was 73% ± 11.4% (*n* = 15). The 1-year OS was 100% (*n* = 20, Supplemental Figure 10). No known harm from the GD treatment was seen. In summary, we found no differences in the rates of diarrhea, aGVHD, graft failure, relapse and relapse-free survival, or OS at 1 year in GD-treated individuals versus no-GD in our study.

### Engraftment and immune reconstitution.

**Figure 4:**
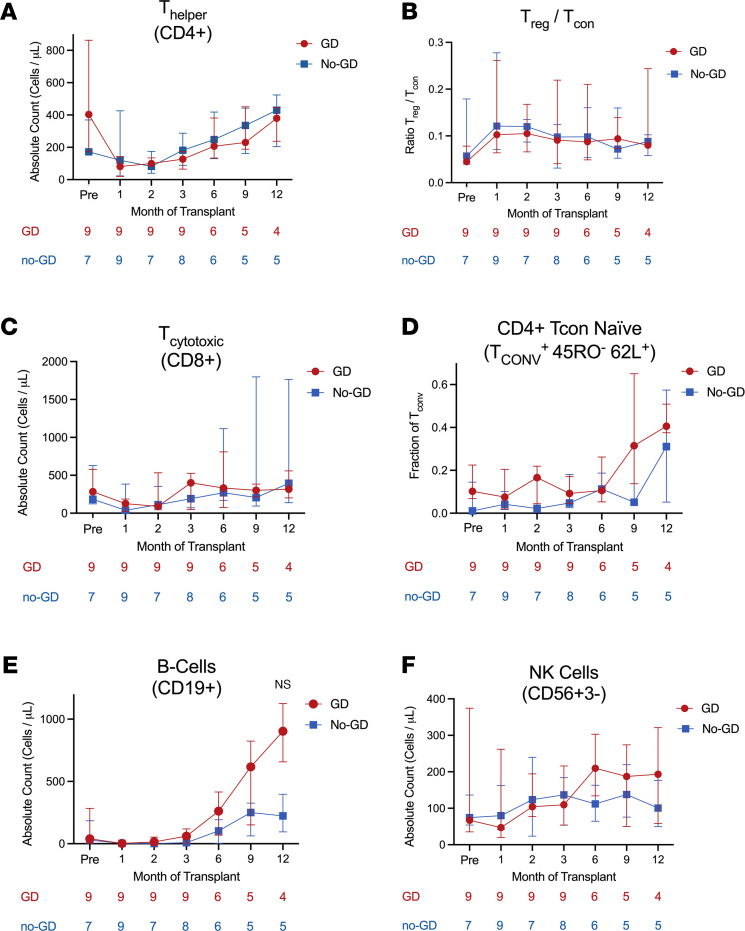
Immune reconstitution. Peripheral blood samples at pretransplant, monthly for the first 3 months, and then months 6, 9, and 12. Shown are the median values ± interquartile range, along with the number of patients sampled at each time point below each graph. (**A**) CD4^+^ T helper cells, (**B**) Treg/T_con_, (**C**) CD8^+^ cytotoxic T cells, (**D**) CD4^+^ T_con_ naive cells, (**E**) CD19^+^ B cells, (**F**) CD56^+^CD3^–^ natural killer cells. For CD19^+^ B cells at 12 months, an uncorrected *P* = 0.016 with a Wilcoxon rank-sum test was not significant when tested against a stringent Bonferroni-adjusted α level of 0.0045 (0.05/11 biomarkers tested).

The median time to neutrophil engraftment was 26 days (IQR, 23.5–29.2) in the GD arm and 24 days (IQR, 19.2–28.0) in the no-GD arm (*P* = 0.47). One patient in the no-GD arm had primary graft failure and underwent a second transplant on day +79. One patient in the GD arm had secondary graft failure and came off the study at day +54. In a prespecified secondary analysis, we examined reconstitution of peripheral blood lymphocyte subsets in the 2 arms, excluding the 2 patients with graft failure. The following analyses of T cell reconstitution revealed similar outcomes in both groups: median CD4^+^ T cell count at 3 months after HCT (126.5 in GD arm vs. 182.1 cells/μL in no-GD arm; *P* = 0.24; Figure 4A); median CD4^+^ T cell count at 6 months after HCT (median, 206.5 in GD arm vs. 248.2 cells/μL in no-GD arm; *P* = 0.70; Figure 4A); ratio of regulatory T cells to conventional T cells (Treg/T_con_) in the 12 months post-HCT (Figure 4B); and naive T cell fraction within CD4^+^ T_con_ (Figure 4D). Taken together, these findings suggest that GD does not adversely affect thymic function. Recovery of CD8^+^ T cells and natural killer cells was similar between the study arms (Figure 4, C and F, respectively). CD19^+^ B cell concentration at 12 months was a median of 903.5 cells/μL (IQR, 814.0–984.8) in the GD arm compared with a median of 223.9 cells/μL (IQR, 190.1–333.3) in the no-GD arm (uncorrected *P* = 0.016; not significant after a stringent Bonferroni-adjusted α level of 0.0045 given 11 biomarkers were tested) (Figure 4E). Of note, none of the patients in the no-GD arm received rituximab as part of their conditioning regimens or as part of posttransplant therapy. While it is difficult to draw conclusions from this small study, future, larger studies may further illuminate if and how immune reconstitution is influenced by GD and the microbiota.

### Incidence of BSI.

**Figure 5:**
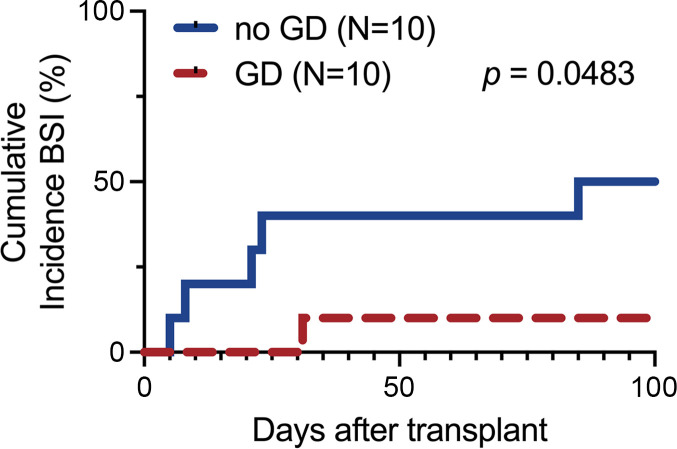
Cumulative incidence of BSI during the first 100 days of transplant. Patients are separated by treatment group with GD (dashed red line, *n* = 10 patients) and no-GD (solid blue line, *n* = 10 patients). In the 6 patients with a BSI, 5 BSIs occurred within the first 31 days; 1 patient in the no-GD arm had BSI on day +85 relative to the first transplant (on day +6 of the second transplant). *P* = 0.0483, Gray’s test.

In an exploratory analysis, we noted a trend of fewer BSIs in patients enrolled in the GD compared with the no-GD arm. During the 100-day study period, a total of 9 BSI episodes occurred in 6 patients, 8 in the no-GD arm and 1 in the GD arm (*P* = 0.09, Table 2 and Supplemental Table 2). Five of the 6 patients had a BSI within the first 31 days. In a post hoc exploratory comparison, the cumulative incidence of BSI was higher in the no-GD arm compared with GD arm (Figure 5, *P* = 0.0483, Gray’s test). Seven of the 9 (78% of total) BSI episodes were from the no-GD arm and occurred before the day of neutrophil engraftment.

### Species-level evidence that the BSI-causing bacterium is present in the gut microbiome.

Given the interesting trend of increased BSI incidence in the no-GD versus GD arm, we hypothesized that GD may decrease the burden of pathogens in the gut microbiota that can translocate across the mucosal barrier and subsequently cause a BSI. The gut microbiota can be a reservoir of BSI-causing pathogens in this patient population (16, 19, 26, 36, 37); thus, we asked whether patients in the no-GD arm had BSI-causing pathogens within their gut microbiota before or during the time of infection.

**Figure 6:**
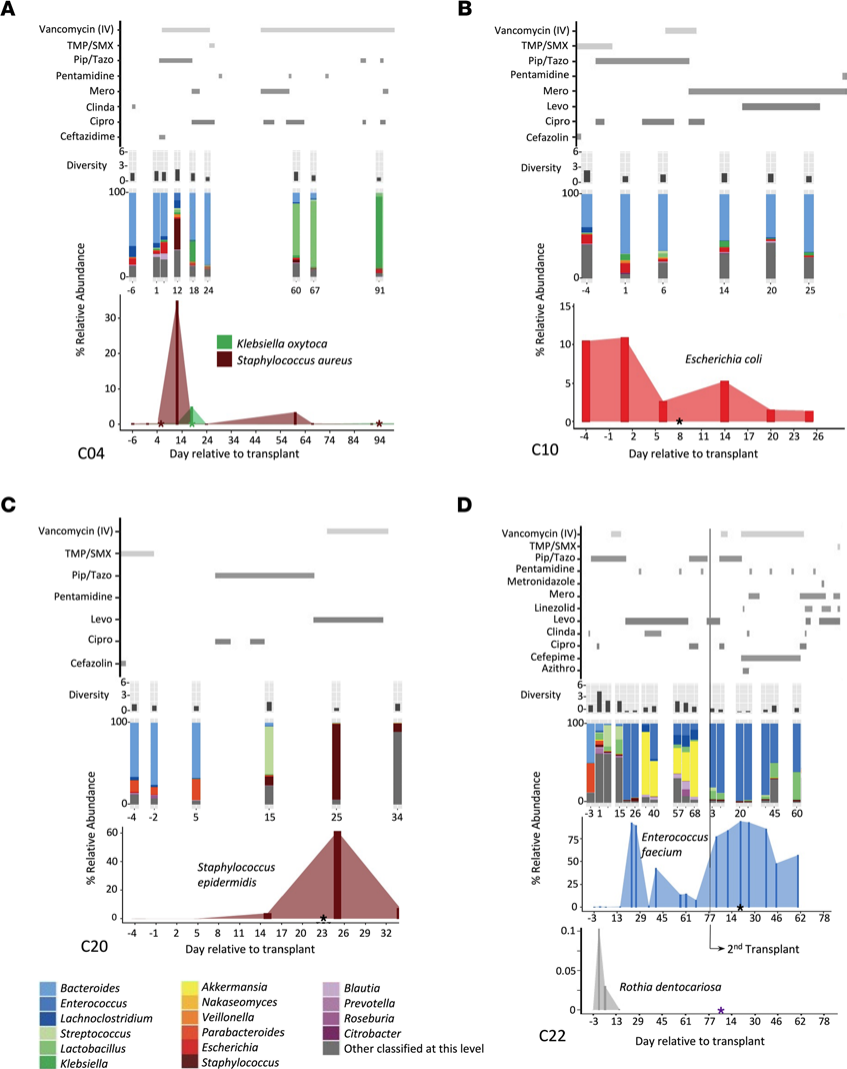
Bacterial abundance in the gut around the time of BSI. Multiple pathogens are present in the gut around the time of the BSI. Days relative to the course of the transplant shown on the *x* axis (from top to bottom on the *y* axis), antibiotic administration, α (Shannon) diversity, relative abundance of microbes in the stool samples at the genus taxonomic level (with organisms listed by color according to the key at the lower left), and relative abundance in the gut of the BSI-causing organism with the date of the BSI shown as an asterisk (*). Note: *y* axis is a different scale between abundance plots for focused organisms found in the BSI. (**A**) Patient C04 had 2 *Staphylococcus aureus* BSIs 89 days apart (day +5 and day +94) and a *Klebsiella oxytoca* BSI on day +18. (**B**) *E*. *coli* BSI on day +8 in patient C10. (**C**) *Staphylococcus epidermidis* BSI on day +23 in patient C20. (**D**) Patient C22 received 2 transplants and had low abundance of *Rothia dentrocariosa* in the gut during the first transplant; *Rothia* was not detectable in the gut after day +15 of the first transplant, with a *Rothia* BSI on day +6 of the second transplant (day +85 relative to the first transplant). An *Enterococcus faecium* BSI occurred on day +20 of the second transplant (day +99 relative to the first transplant). Antifungal and antiviral medications are shown in Supplemental Figure 13. Information on patients C03 and C11 may be found in Supplemental Figure 11. Azithro, azithromycin; Cipro, ciprofloxacin; Clinda, clindamycin; Levo, levofloxacin; Mero, meropenem; PipTazo, piperacillin/tazobactam; TMP/SMX, trimethoprim/sulfamethoxazole (cotrimoxazole).

In 7 of 9 of the BSI episodes, we were able to identify the BSI species in the gut (relative abundance > 0.1%) within 4 days of the BSI (Figure 6 and Supplemental Figure 11; patients C03, C04 [2 of 3 BSIs], C10, C11, C20, C22 [1 of 2 BSIs]). We observed typically enteric bacteria causing a BSI in 3 patients (*Klebsiella oxytoca*, *K. oxytoca*, in patient C04; *E. coli* in C10; and *E. faecium* in C22). In addition, we observed increasing relative abundance of *Leclercia adecarboxylata* (*L. adecarboxylata*) in the gut prior to the BSI in patient C03 (Supplemental Figure 11A). Interestingly, 3 independent BSI episodes were caused by organisms in the genus *Staphylococcus*, which is typically categorized as nonenteric, non-MBI related (38). In each of these cases, *Staphylococcus* was found in the intestinal microbiota within 4 days of the BSI episode (Figure 6, A and C; patient C04, who had 2 independent BSIs 89 days apart at 35% and 0.1% relative abundance, and C20 at 61% relative abundance). By contrast, we detected *Staphylococcus aureus* (*S. aureus*) in fewer than 0.005% of the total reads in stool samples from 3 independent healthy controls (Supplemental Figure 4).

Two of the 9 BSI episodes (a *Bacillus* bacterium and *Rothia* *dentocariosa*, *R.* *dentocariosa*) are less likely to be derived from the lower gut (Supplemental Figure 11B and Figure 6D). While there was a rise in the relative abundance of the genus *Bacillus* in the stool sample (Supplemental Figure 11B, patient C11), the BSI isolate from C11 was subsequently sequenced and found to be *Lysinibacillus fusiformis* (*L. fusiformis*, also called *Bacillus fusiformis*, Supplemental Table 2), which was not detected in any of the stool samples of this patient. This suggests that either the BSI did not originate from the distal gut or the BSI was low enough in abundance to preclude taxonomic identification at the species level. The typically oral microbe *R.* *dentocariosa* was detectable at a low abundance in the fecal sample early in the first transplant of patient C22 and was undetectable after day +15, suggesting this BSI either originated from a location other than the distal gut or was in low abundance in the lower gastrointestinal tract at the time of the BSI (Figure 6D).

### Strain-level evidence that multiple BSI isolates are identical or nearly identical to those found in the gut microbiota around the time of the BSI.

In the previous analysis, we found temporal concordance between species in the gut microbiota and the subsequent BSI, which suggested possible bacterial translocation across a damaged intestinal epithelium. If the BSI and gut strain were identical, this would support the gut being a likely source of infection. Thus, we looked for the presence of concordant strains of the BSI-causing pathogens within the gut. To carry out this strain analysis, we used *inStrain* (30), a tool that can account for multiple strain populations in metagenomic sequencing data. Specifically, the population average nucleotide identity (popANI) metric only calls single nucleotide polymorphisms (SNPs) in positions where 2 samples do not share any alleles (30). For example, 2 organisms of the same bacterial species will share >95% ANI (5 mismatches for every 100 bases compared) (30); 2 microbes are considered nearly identical if they have >99.9999% ANI (1 mismatch for every 1 million bases compared).

For this analysis, we generated whole genome sequencing data from 11 different BSI isolates from 9 BSI episodes (Supplemental Tables 2 and 6); all strains analyzed were from no-GD patients except for 1 strain from patient C11 from the GD arm. A median of 32.4 × 10^6^ (range, 26.4 × 10^6^ to 46.6 × 10^6^) reads were generated for each BSI isolate, and 15.6 × 10^6^ (range, 7.5 × 10^6^ to 23.5 × 10^6^) reads remained after preprocessing (estimated median 545-fold genome coverage). Draft genomes from BSI organisms were assembled and used as references for the *inStrain* comparison (assembly statistics in Supplemental Table 7). Sequencing reads from all stool and BSI isolates were mapped against a patient’s BSI draft genome, and samples with at least 20,000 mapping reads were retained. SNPs were called with *inStrain profile*, and pairs of samples were compared with *inStrain* *compare*. Pairs of samples that had at least 50% of the genome covered at a depth of at least 5 reads were considered for further analysis.

Going through the most notable cases individually (Table 3), no-GD patient C04 had 2 independent *Staphylococcus aureus* BSIs on day +5 and day +94 (Figure 6A). The *S*. *aureus* in the stool sample from day +12 was identical to the BSI on day +5 (100% popANI, 0 SNPs detected in 2.8 Mb of sequence compared [the size of the *S*. *aureus* genome is 2.8 Mb]; Table 3 and Supplemental Figure 12A). The *S*. *aureus* BSI on day +94 was nearly identical to the stool sample on day +12 (99.9999% popANI; 2 SNPs between samples). Patient C04 also had detectable *K. oxytoca* in the stool (0.3% relative abundance on day +12; 5% on day +18) and had a BSI with the same strain of *K*. *oxytoca* (100% popANI with 6 Mb compared [the *K*. *oxytoca* genome is 6.02 Mb]) on day +18 (Figure 6A). However, as *Klebsiella* was in lower abundance and not sequenced to high enough depth in samples prior to day +18, we were unable to determine whether the strain was present prior to the BSI. Interestingly, while the clinical microbiology lab identified that the 2 *Klebsiella* strains from the blood culture had different sensitivity to ceftriaxone (Supplemental Table 6), the 2 isolates were identical by *inStrain* (100% popANI, 0 SNPs) at the genomic level.

Patient C10 in the no-GD arm had an *E*. *coli* BSI on day +8 (Figure 6B). An identical (100% popANI, 5 Mb compared [the *E*. *coli* genome is 5.12 Mb]) strain was found in the 6 stool samples collected from days +1 to +32. However, a different *E*. *coli* strain was present in the stool at day –4 (99.7% popANI to BSI and other stool samples, with approximately 13,303 different SNPs, Table 3 and Supplemental Figure 12B). Thus, at least 2 strains of *E*. *coli* were observed in this patient at the different time points. It is possible that the BSI-causing strain was present at day –4 but was simply below our limit of detection in this earlier sample. The 2 *E*. *coli* BSI samples on the same day (+8) from the clinical microbiology laboratory (Supplemental Table 6) were nearly identical to each other, with 1 SNP detected (99.9999% popANI).

Patient C22 in the no-GD arm experienced 2 BSIs after a second transplant (Figure 6D; *R. dentocariosa* on day +6 and *E. faecium* on day +20, relative to second transplant). In the 18 stool samples from this patient, *R*. *dentocariosa* achieved a maximum of 0.1% relative abundance without enough sequencing coverage to conduct an *inStrain* comparison. By contrast, *E*. *faecium* in patient C22 was at more than 92% relative abundance at the time of the *E*. *faecium* BSI (Figure 6D). In the samples from day +23 of the first transplant through the end of the study (14 samples total), *E*. *faecium* in the gut was nearly identical (>99.9999% popANI) to the BSI strain, suggesting that the same strain was present in this patient’s microbiota through the course of 2 transplants and likely eventually caused BSI (Figure 6D, Table 3, and Supplemental Figure 11C).

In patients C03 (no-GD arm) and C11 (GD arm), the organism (*L. adecarboxylata* and *L. fusiformis*, in C03 and C11, respectively) found in the BSI was either undetectable or at low abundance and did not have sufficient sequencing depth and coverage in the stool samples to make a conclusion regarding strain specificity using the *inStrain* comparison (Table 3). No-GD patient C20 experienced a *Staphylococcus epidermidis* (*S. epidermidis*) BSI on day +23, with a rise in relative abundance in the gut microbiota from 3.7% on day +15 to 61% on day +25 (Figure 6C), suggesting the BSI originated from the gut; however, we were unable to do strain-level analysis as the original BSI-causing isolate was archived, but upon sequencing was identified to be *E*. *coli*, a likely contaminant of the culture (Table 3).

Based on these findings, at least 5 of the 9 BSIs are identical or nearly identical to a species found in the gut microbiota using the popANI genome comparison of *inStrain*. Two additional BSIs (*L. adecarboxylata* from patient C03 and *S. epidermidis* from patient C20) may also have originated from the gut based on an increase in the relative abundance of the bacteria around the time of the BSI, although strain-level confirmation of this prediction is lacking. This suggests that in up to 7 of the 9 BSIs, the infection-causing pathogen is present in the gut in patients from the no-GD arm; by contrast, none of the BSI-causing pathogens are present in the gut in patients from the GD arm (Figure 6 and Supplemental Figures 11 and 12). Collectively, these data demonstrate that the gut microbiota is a reservoir for pathogens traditionally derived from the gut and that microbes like *Staphylococcus*, which are not typical gut bacteria, may subsequently cause a gut-derived BSI in allo-HCT patients.

## Discussion

Due to the lack of evidence supporting a clear benefit, GD is not recommended as a standard practice for the prevention of aGVHD or bacteremia. An informal survey of transplant centers in the United States in 2017 indicated that approximately 40% of adult and pediatric centers routinely practiced GD (7). As part of this study, we surveyed 101 pediatric HCT centers in the United States and Canada regarding their GD practices in 2019. Of the 32 centers that responded, only a small proportion (3 of 32; 9.4%) of centers were using GD as part of the aGVHD prophylaxis regimen (Supplemental Data 1). The practice of GD in patients undergoing allo-HCT is based on murine data, as well as early single-arm and retrospective studies suggest that using GD to alter the intestinal microbiota may protect against aGVHD (2, 5, 8). In this study, probably the first prospective randomized trial of GD in allo-HCT recipients, we investigated how GD with oral vancomycin-polymyxin B alters the microbial composition on a species and strain level using NGS. We also characterized secondary clinical outcomes and identified BSI-causing pathogens traceable to the gut either temporally or by strain-specific comparative genomics.

We show that there is no appreciable difference in gut microbiome Shannon diversity during the peritransplant period between GD and no-GD arms. While patients in the GD arm had variable administration of vancomycin-polymyxin B, the changes in diversity at 2 weeks did not correspond to the amount of GD the individual received. Furthermore, GD does not appear to lead to a decreased use of systemic antibiotics, as the time of exposure to prophylactic and therapeutic antibiotics was similar between the 2 arms. Thus, the equivalent exposure to systemic antibiotics in the 2 arms may have masked the impact of GD on Shannon diversity within the gut microbiome. Alternatively, while Shannon diversity is sensitive to loss of rare taxa (34), a different analysis with a future larger study, such as a mixed-effect linear model, may be able to evaluate if there is a differential abundance of species between the GD and no-GD arms.

Despite promising evidence from earlier studies indicating that nonabsorbable antibiotics were associated with a lower incidence of GVHD (1, 5), recent data suggest a more complex picture. For example, 1 retrospective report suggests that choice of antibiotics is critical, as cefuroxime, tobramycin, and nystatin in the GD arm were associated with an increased risk of developing aGVHD compared with no-GD (39). Several studies have expanded this concept, showing that systemic broad-spectrum antibiotics and loss of microbial diversity (specifically commensal organisms) are associated with gastrointestinal GVHD and GVHD-related mortality (10, 31). While our study is not powered to assess a difference in aGVHD incidence, we found that 3 patients had grade III–IV aGVHD in the no-GD arm versus 1 patient in the GD arm with grade II aGVHD. However, this trend could be attributed to the higher number of matched unrelated donors in the no-GD arm than the GD arm (*n* = 5 vs. *n* = 2, respectively). Collectively these data leave an open question as to whether GD decreases the risk of grade III–IV aGVHD.

Based on prior studies suggesting interactions between the gut microbiota and development of circulating immune cells, we examined immune reconstitution of T and B cell subsets over the first year post-HCT. Although the reconstitution of T cell and B cell subsets was similar between the study arms after a Bonferroni correction, there was an interesting trend in the absolute CD19^+^ B cell count at 12 months between the arms (Figure 4E). This trend cannot be explained by in vivo depletion as none of the patients in the no-GD arm received rituximab after HCT. Several reports demonstrate extensive crosstalk between the microbiota and B cell diversity (40–42) and that early B lineage development in mice is influenced by the gut microbiome (43). While it is difficult to draw inferences with the small numbers here, an analysis of immune reconstitution and potential implications for infection risk should be fully characterized in a larger study.

While the impact of GD on infection-related outcomes was not part of our prespecified primary or secondary analyses, we postulated that GD with oral vancomycin-polymyxin B may have an impact on decreasing the rate of BSIs that originated from the gut compared with no-GD (Supplemental Figure 14 and Supplemental Data 4). In an exploratory analysis, we found that all BSIs where the pathogen was found concomitantly in the gut were observed in the no-GD arm (BSIs did not appear dependent upon systemic corticosteroid therapy as 3 of 9 BSI episodes had concurrent corticosteroid therapy at the time of BSI). Interestingly, historical publications reporting on GD demonstrated that many of the Gram-negative bacteria isolated from the BSIs were still sensitive to the drugs used for decontamination (22), and a meta-analysis of ICU patients showed no increase in antimicrobial resistance with selective decontamination (44). The primary mechanism of GD reducing bacteremia in critical care patients is thought to be through limiting the growth of select bacteria, including bacteria such as *Enterococcus* and from the phylum Proteobacteria (45), which includes the genera *Escherichia* and *Stenotrophomonas*. No prospective study in pediatrics has been conducted to date to our knowledge with a standard therapy without GD arm that is appropriately powered to address if GD decreases the risk of BSI. Furthermore, there have been very limited studies to compare systemic antibiotic prophylaxis and nonabsorbable GD (including ref. 46 and a meta-analysis: ref. 47). The largest pediatric study to demonstrate a reduction in bacteremia was conducted in children undergoing initial therapy for acute leukemia and used levofloxacin for systemic prophylaxis, rather than gut decontamination; a second arm of the study did not reach the level of statistical significance for children undergoing HCT (48). Given the above findings, there are insufficient data supporting a clear benefit of GD in humans in HCT at this time. However, a larger trial may inform the question of whether oral vancomycin-polymyxin B decreases the burden of pathogens in the gut microbiota that can then translocate across the mucosal barrier and subsequently cause a BSI.

HCT patients often have injured mucosae secondary to conditioning chemotherapy and neutropenia, increasing their risk of mucosal barrier injury laboratory-confirmed bloodstream infection (MBI-LCBI). Due to their immunocompromised status and central venous catheters, HCT patients are also at risk for non–MBI-LCBI infections, including central line–associated BSI (CLABSIs). This is an important distinction as MBI-LCBIs are not prevented by improved central venous catheter care when compared to CLABSIs (12, 49–51). Given that there is approximately 18% mortality attributable to BSI in this patient population (12), being able to identify and develop methods to decrease this rate could have a profound impact on patient survival during HCT. We demonstrate here that the bacterial species responsible for the BSI are found in the gut in 7 of 9 BSI episodes. While many of those identified in the gut microbiome are Gram-negative enteric organisms, we also found evidence of classically non–MBI-LCBI organisms, such as *Staphylococcus* species. These data, along with previous work (19, 26, 36, 37, 52), strongly suggest that the gut can serve as a reservoir for pathogens in HCT recipients. Overall, there may be skin- and nares-resident organisms such as *S*. *aureus* and *S*. *epidermidis* that can colonize and grow to high relative abundance in the gut microbiomes of severely immunocompromised hosts. Furthermore, while our study focused on young patients, it is possible that reduction of gut-derived BSI may be more relevant for improving HCT outcomes in older patients.

While the findings presented here suggest that GD is generally safe and has a relatively limited impact on the gut microbiome, there are several limitations to our study. First, this is a single-center study with a small sample size. While originally powered to detect a difference of gut microbiome Shannon diversity of 4.0 in the no-GD arm and 2.8 in the GD arm based on changes in adult HCT patients (16), the magnitude of the change was less than anticipated. Some of this disparity is likely due to confounding variables such as systemic antibiotics creating an even larger heterogeneity of the microbiota data than originally anticipated. Poor adherence to the oral vancomycin-polymyxin B regimen, likely due to palatability, also may have lessened the effect between the arms. We also do not consider the microbiota from other sites in the body (e.g., skin) and thus cannot definitively exclude the skin or other sites as the source of the BSI.

Despite these limitations, the prospective design of this study, the standardized collection of samples, the application of NGS technologies, and the use of *inStrain* to compare BSI genomes and fecal metagenome assemblies (MAGs) enable us to investigate the impact of GD versus no-GD at a detailed level. In particular, whole-genome sequencing of stool and bacterial isolates, along with use of the *inStrain* algorithm, allows for the confident and accurate comparison of bacterial isolates and metagenomes to identify identical or nearly identical microbial strains. In addition, by using a genome-wide comparison, we provide the framework to subsequently interrogate strain specificity, which can have important impacts on antibiotic resistance and pathogenicity of particular organisms (e.g., exploiting metabolic pathways or secreted proteins of specific strains).

A larger study could test the concept that GD decreases the number of, or eliminates entirely, bacteria that can potentially be pathogenic (Supplemental Figure 14) and translocate across the mucosal barrier. In addition, bacteria that caused the infections may be dependent on other bacteria that are susceptible to vancomycin-polymyxin B. Microbes in the gut live in communities, and when keystone species are lost, it may lead to fundamental changes in the community through a “cascading” effect. While efforts have been made to use the microbiota composition to predict the risk of a BSI (18, 20, 21), it remains an open question whether targeting of microbes can be achieved to decrease the risk for BSI in the allo-HCT population (53). In conclusion, in this phase II randomized controlled trial of 20 patients, we noted that all BSIs traced to the gut were found in the no-GD arm. Furthermore, *Staphylococcus* was found in the gut, suggesting that expanding the number of organisms defined as MBI-LCBI for allo-HCT patients is an important step for detecting and subsequently designing ways to mitigate the risk of BSI during HCT. Finally, these data suggest that oral vancomycin-polymyxin B GD may protect against post-HCT BSI by decreasing the prevalence or abundance of pathogens that can translocate across the mucosal barrier and subsequently cause gut-derived BSIs, a finding that will need to be verified in a larger trial.

## Methods

### Cohort selection and study design

We performed a randomized phase II trial (±GD, ClinicalTrials.gov identifier: NCT02641236, CONSORT diagram in Figure 1) examining the impact of GD with oral vancomycin-polymyxin B on intestinal microbiota composition of allo-HCT patients compared with no-GD. Eligibility included any recipient, ages ≥4 years to 30 years (adult: 18–30 years, pediatric: 4–17 years) and toilet trained, of 9/10 or 10/10 matched bone marrow allo-HCT, or 4/6, 5/6, and 6/6 matched cord blood allogeneic HCT. Stools from 2 healthy sibling donors were collected as a comparison group (ages ≥4 years and toilet trained). Detailed eligibility criteria and CONSORT checklist are available in Supplemental Data 2 and Supplemental Data 3. Enrolled patients were randomized (1-to-1) to either “GD” or “no-GD.” The primary endpoint was microbial (Shannon) diversity at 2 weeks after HCT. The trial was powered to detect a difference in Shannon diversity index of 1.2 (4.0 for no-GD vs. 2.8 for GD) with a 1-sided *t* test and α = 0.05, and assuming a standard deviation of 0.9 based on a previous study (16). An intention-to-treat comparison of GD versus no-GD was performed using the Wilcoxon rank-sum test. Participants assigned to the GD arm received nonabsorbable, oral vancomycin-polymyxin B capsules according to body surface area (BSA): 375 mg/m^2^ BSA of vancomycin and 187 mg/m^2^ of polymyxin B (see Supplemental Table 1 for details). Random allocation of anonymous identifier, enrollment, and assignment were completed under the supervision of the principal investigator of the clinical trial. Oral GD began on day –5 relative to the hematopoietic cell infusion date (day 0) and continued through neutrophil engraftment, defined as an absolute neutrophil count ≥ 500 cells/mm^3^ for 3 consecutive days. Patients in no-GD received the institutional standard practice including all other supportive care as did patients in the GD arm. Antifungal and antiviral prophylaxis was used at the discretion of the treating physician generally starting at day –9 (e.g., fluconazole) and day –5 (e.g., acyclovir), respectively (administration data in Supplemental Figure 13). Use of any agent (e.g., sulfamethoxazole/trimethoprim, pentamidine) for prophylaxis of *Pneumocystis jirovecii* pneumonia was permitted. Secondary endpoints include the frequency of diarrhea (>3 stools per day) in the first 7 days after HCT, incidence of grade ≥II aGVHD during the first 100 days posttransplant, survival, malignant disease relapse at 1 year after study entry, progression-free survival (defined as time from randomization to the earlier of progression of malignant disease or death due to any cause at 1 year after study entry), and immune reconstitution. Exploratory outcomes include bacteremia during the first 100 days posttransplant. Clinical records including details of the transplant type, conditioning regimen and prophylaxis medications for aGVHD, antibiotic administration, microbiological information (including BSI and antibiotic resistance data), clinical symptoms (including aGVHD and diarrhea), and outcomes (relapse, death, and aGVHD) were obtained from the patient chart.

Stool samples were collected and stored immediately at 4°C and frozen at –80°C in cryovials within 24 hours of collection. Stool samples were collected as follows: weekly (±3 days) prior to neutrophil engraftment; monthly (±2 weeks) after neutrophil engraftment until 6 months; at 6 months and 1 year (±1 month); and within 48 hours of aGVHD diagnosis or positive blood culture.

### Flow cytometry and immune profiling

Phenotypic analyses of lymphocyte subsets were performed at pretransplant and 1, 2, 3, 6, 9, and 12 months after transplant. Briefly, 50 μL of EDTA whole blood was subjected to red blood cell lysis using 1× BD-PharmLyse (BD Biosciences) and subsequently incubated with fluorochrome-conjugated monoclonal antibodies (Supplemental Table 10) with individual subsets enumerated in a FACSCanto II flow cytometer and analyzed using BD FACSDiva (both from BD Biosciences) and FlowJo software (TreeStar) as described previously (54). CD4^+^ Tregs were defined as CD3^+^CD4^+^CD25^med-hi^CD127^lo^, CD4^+^ T_con_ as CD3^+^CD4^+^CD25^neg-lo ^CD127^med-hi^, B cells as CD19^+^, cytotoxic T cells as CD8^+^, and natural killer cells as CD56^+^CD3^–^. Within CD4^+^ Tregs and CD4^+^ T_con_, subsets were defined as follows: naive T cells (CD45RO^–^CD62L^+^), central memory (CD45RO^+^CD62L^+^), and effector memory (CD45RO^+^CD62L^–^).

### BSI antibiotic susceptibility testing

Antibiotic susceptibility testing on isolates from bloodstream infections was performed by the Clinical Microbiology Laboratory at BCH/Dana-Farber Cancer Institute (DFCI), except for colistin (polymyxin E), which was performed at Stanford Health Care Clinical Microbiology Laboratory using the disk elution test as previously described (55). Minimal inhibitory concentrations for Enterobacteriales were interpreted using breakpoints according to Clinical and Laboratory Standards Institute (56) and colistin (polymyxin E) using the European Committee on Antimicrobial Susceptibility Testing (57).

### BSI isolate cultures

BSI isolates from HCT patients were obtained from the Clinical Microbiology Laboratory at DFCI. Isolates were grown on agar slants, transferred to Luria-Bertani broth, and grown to saturation at 37°C. Bacteria were pelleted by hard spin (10,000*g*) followed by removal of the supernatant and frozen at –80°C until DNA extraction.

### DNA extraction and WGS metagenomic sequencing of stool samples and BSI isolates

Genomic DNA was extracted from stool samples and BSI cultures using the QIAamp Fast DNA Stool Mini Kit (QIAGEN, catalog 19593) per the manufacturer’s instructions with the following modifications: in suspension buffer, samples were heated to 95°C and subjected to 7 rounds of bead-beating for 30 seconds, alternating with cooling on ice for 30 seconds prior to addition of proteinase K and lysis buffer. DNA concentration was measured using Qubit Fluorometric Quantitation (DS DNA High-Sensitivity Kit, catalog Q32851, Thermo Fisher Scientific). DNA sequencing libraries were prepared using the Nextera XT DNA Library Prep Kit (Illumina) and Nextera FLEX (Illumina) for samples unable to be prepared with Nextera XT due to low biomass. DNA library fragment length distributions were quantified via Bioanalyzer 2100 instrument (Agilent Technologies) using the High Sensitivity DNA kit (catalog 5067-4626, Agilent Technologies) per the manufacturer’s protocol.

Libraries were pooled with unique dual sample indices to avoid barcode swapping (58) and sequenced on Illumina HiSeq4000 or NovaSeq P150 platforms with a read length of 150 bp. Sequencing was performed by Novogene.

For microbiota analysis, a healthy donor stool was used as a positive control for batch-to-batch variation, a known microbiota community (ZymoBIOMICS microbial community standard, catalog D6300) to determine microbial extraction bias, and a negative control carried through the entire DNA extraction, library preparation, and sequencing.

### Computational methods

#### Preprocessing.

Fecal and BSI short-read WGS metagenomic sequencing reads were preprocessed to remove sequencing adapters, PCR artifacts and duplicate reads, and any reads mapping to the human genome, using established bhattlab_workflows available at GitHub, commit ID d11b146, https://github.com/bhattlab/bhattlab_workflows/blob/master/manual/preprocessing.md (59). Briefly, sequenced reads were deduplicated using SuperDeduper (60) and trimmed using TrimGalore! v0.6.5 (61) with a minimum quality score of 30 for trimming and minimum read length of 60. All reads that aligned to the human genome (hg19) were removed using BWA v0.7.17 (62) with final results of preprocessing read counts shown in Supplemental Figure 3. Sequences then underwent quality control using FastQC v0.11.9 (63). Bioinformatics workflows were implemented using Snakemake (64).

#### Classification with Kraken2 and diversity calculations.

Short-read data was taxonomically classified using Kraken2 (32) against a database of all bacterial, fungal, and viral genomes in the NCBI GenBank database assembled to complete genome, chromosome, or scaffold quality as of January 2020. Species abundance was estimated using the Bracken (65) database, built using a read length of 150 and *k-mer* length of 35. Kraken2 classification workflows are available at GitHub: commit ID dd2928e, https://github.com/bhattlab/kraken2_classification (66). Diversity of the microbes was calculated using Vegan v2.5-7 (67).

#### Assembly and binning.

Short-read sequences from stool samples and BSI isolates were assembled using SPAdes v3.15.2 (68). Stool metagenomic sequences were subsequently binned using CONCOCT v1.1.0 (69), MetaBAT 2 v2.15 (70), and MaxBin v2.2.7 (71); aggregated using DASTool v1.1.1 (72); and dereplicated using dRep v2.5.4 (73). Bins were evaluated for completeness and contamination using QUAST (74). MAG quality was assessed using previously established standards by Bowers et al. (75) and Nayfach et al. (76). Workflows are available at GitHub, commit ID d11b146, https://github.com/bhattlab/bhattlab_workflows/blob/master/manual/assembly.md (59).

#### Antibiotic resistance gene detection.

Assembled BSI contigs and binned contigs from stool metagenomic sequences were profiled for antibiotic resistance genes with the Comprehensive Antibiotic Resistance Database and the Resistance Gene Identifier using default parameters (77). Known colistin (polymyxin E) resistance genes detected in the assembly of Gram-negative BSIs are listed in Supplemental Table 9.

#### Determining strain specificity of BSI isolates and stool metagenome assemblies.

To compare bacterial strains and gut metagenomes in multiple samples, we used *inStrain* v1.0.0 (30). Sequencing reads were mapped against assembled BSI genomes using BWA (62). Pairs of samples with more than 50% coverage breadth at a depth of at least 5 reads were compared to analyze SNPs and determine ANI between the samples.

#### Data availability.

All sequencing data sets from the current study have been deposited in the Sequence Read Archive under the NCBI BioProject ID PRJNA787952 at http://www.ncbi.nlm.nih.gov/bioproject/787952

### Statistics

Taxonomic abundance plots, antibiotic time course, and vancomycin-polymyxin B dosage graphs were created using GraphPad Prism version 9.1.2 for MacOS, GraphPad Software, and the ggplot2 package v3.3.3 (78) with code modified from previous reports (19, 26, 79). Comparisons by treatment group were performed using Fisher’s exact test (for binary variables), Wilcoxon rank-sum test (for continuous variables), or Wilcoxon signed-rank test (for paired continuous variables) for comparison of baseline versus 2 weeks after HSCT within patients. The Wilcoxon rank-sum and Wilcoxon signed-rank tests were adjusted using an FDR of 0.05 or Bonferroni correction. Cumulative incidence curves of BSI were compared using the Gray’s test with adjustment for the competing risk of death. We calculated α and β diversity using the vegan package v2.5-7 (67) and compared with the Wilcoxon rank-sum test and corrected using an FDR ≤ 0.05. ANOSIM statistic after 999 permutations was done for comparison of β diversity for patients with healthy sibling samples to compare. Figure 2 and graphical abstract were created with BioRender.

### Study approval

The trial was approved by the institutional review board (IRB) of DFCI (Protocol #15-394 approved October 2015; principal investigator: JSW) and was performed at BCH and DFCI. IRB protocol was open to patient entry March 2016 through September 2019. Written informed consent for the patient (if ≥18 years), parent (if <18 years), or legally authorized representative was obtained prior to any specimen collection. Full protocol available at DFCI. Trial is registered under ClinicalTrials.gov identifier NCT02641236.

Prior publication: Interim analyses have been previously reported in abstract form: *Blood*. 2019;134(suppl 1):5665. https://doi.org/10.1182/blood-2019-122480

## Author contributions

JSW, ASB, and JR conceived of the original study. WBL and JSW designed the clinical trial. LEL, SPM, and CND contributed to the clinical protocol and assisted in enrollment and acquisition of clinical samples. S Silverstein, SK, and OB collected the clinical samples. CJS, JSW, WBL, and NC analyzed the raw clinical data from charts and generated tables and figures. CJS and MML extracted DNA, prepared short-read sequencing libraries, and selected samples for sequencing. CJS, BAS, and ASB analyzed the sequencing quality, conceived of the assembly approach, and performed the *inStrain* analysis. CGR. performed flow cytometry and immune profiling. NB and AM performed analysis of antibiotic resistance of Gram-negative BSIs to polymyxin B/colistin. CJS and STJK wrote and modified code and generated figures for the clinical data. CJS and TMA analyzed the infectious disease data. S Sun and AAF modeled and considered alternative analysis paths for the microbial sequencing data sets. CJS wrote the original manuscript and generated figures and tables. All authors reviewed, commented on, and approved the manuscript.

## Supplementary Material

Supplemental data

ICMJE disclosure forms

## Figures and Tables

**Table 1:**
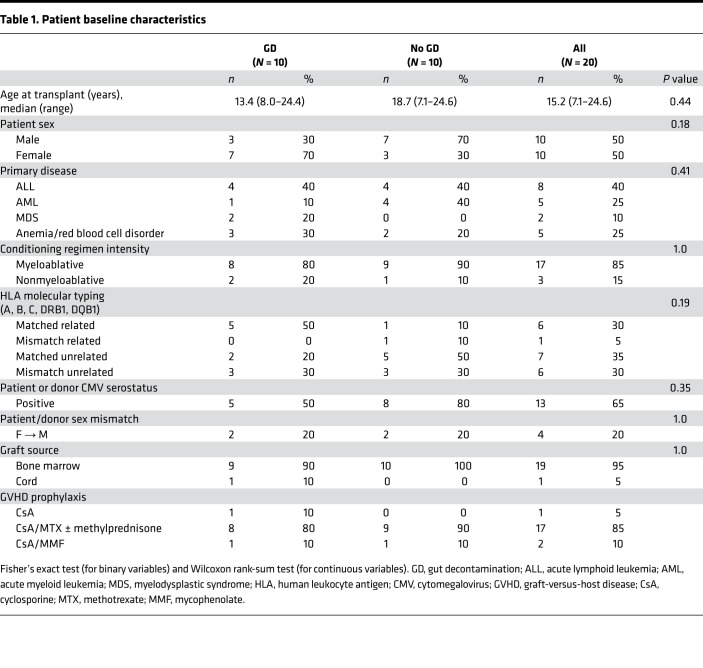
Patient baseline characteristics

**Table 2:**
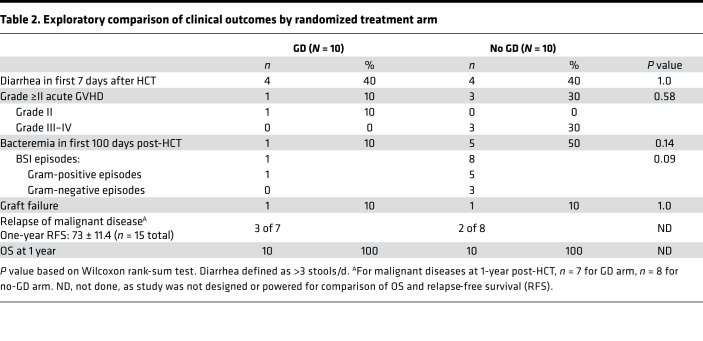
Exploratory comparison of clinical outcomes by randomized treatment arm

**Table 3:**
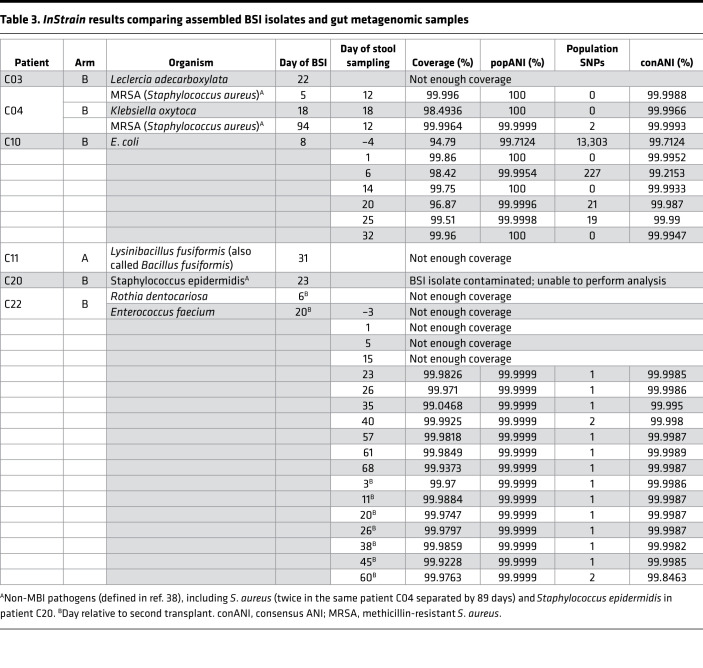
*InStrain* results comparing assembled BSI isolates and gut metagenomic samples

## Version 1 03/03/2022

In-Press Preview

## Version 2 04/08/2022

Electronic publication
