# Supplementary material for: Microbiota dynamics in a randomized trial of gut decontamination during allogeneic hematopoietic cell transplantation
Source: JCI Insight. 2022 Apr 8;7(7):e154344. doi: 10.1172/jci.insight.154344 (PMC9057614; doi:10.1172/jci.insight.154344)
Supplement: Supplemental data [file jciinsight-7-154344-s272.pdf]

Supplementary Materials for

Microbiota dynamics in a randomized trial of gut decontamination during allogeneic hematopoietic cell transplantation

Authors:

Christopher J. Severyn[1], Benjamin A. Siranosian[2], Sandra Tian-Jiao Kong[3], Angel Moreno[4], Michelle M. Li[5], Nan Chen[6], Christine N. Duncan[6,7], Steven P. Margossian[6,7], Leslie E. Lehmann[6,7], Shan Sun[8], Tessa M. Andermann[9], Olga Birbrayer[10], Sophie Silverstein[10], Soomin Kim[10], Niaz Banaei[4,11], Jerome Ritz[7,10], Anthony A. Fodor[8], Wendy B. London[6,7,10], Ami S. Bhatt[2,**] and Jennifer S. Whangbo[6,8,**]

**These authors contributed equally; Co-corresponding Authors.

Affiliations:

[1]Department of Pediatrics, Division of Hematology/Oncology/Stem Cell Transplant and Regenerative Medicine, Stanford University, Palo Alto, CA;

[2]Departments of Genetics and Medicine, Division of Hematology, Stanford University, Stanford, CA;

[3]Department of Biology and Biomedical Informatics, Stanford University, Palo Alto, CA;

[4]Department of Pathology, Stanford University, Palo Alto, CA;

[5]Department of Biomedical Informatics, Harvard Medical School, Boston, MA;

[6]Dana-Farber/Boston Children's Cancer & Blood Disorders Center, Boston, MA;

[7]Harvard Medical School, Boston, MA;

[8]Department of Bioinformatics and Genomics, University of North Carolina at Charlotte, Charlotte, NC;

[9]Department of Medicine, Division of Infectious Diseases, University of North Carolina at Chapel Hill, Chapel Hill, NC;

[10]Dana-Farber Cancer Institute, Boston, MA

[11]Department of Medicine (Infectious Diseases), Stanford University, Palo Alto, CA

ORCID information for authors:

	ORCID
Christopher J. Severyn	0000-0002-0884-6372
Benjamin A. Siranosian	0000-0002-4935-8172
Sandra Tian-Jiao Kong	0000-0002-0044-562X
Angel Moreno	
Michelle M. Li	0000-0003-0223-7485
Nan Chen	
Shan Sun	0000-0003-0349-2664
Tessa M. Andermann	0000-0002-0844-7400
Sophie Silverstein	
Soomin Kim	0000-0001-8938-2559
Olga Birbrayer	
Niaz Banaei	0000-0001-8501-3000
Christine N. Duncan	
Steven P. Margossian	
Leslie E. Lehmann	
Jerome Ritz	0000-0001-5526-4669
Anthony A. Fodor	
Wendy B. London	0000-0003-3571-6538
Ami S. Bhatt	0000-0001-8099-2975
Jennifer S. Whangbo	0000-0002-5759-2532

This file includes:

Table S1 | Vancomycin and Polymyxin B administration details

Table S2 | Bloodstream infections

Table S3 | Shannon diversity at the species and genus taxa of patient samples at baseline (prior to any GD exposure) and two-weeks after transplant

Table S4 | Shannon diversity baseline and 2-week comparisons

Table S5 | Duration of antibiotic exposure within the first 30 days post-HCT

Table S6 | Antibiotic susceptibility testing of blood culture isolates

Table S7 | BSI Reads and Coverage based on estimated Genome size

Table S8 | Colistin (polymyxin E) resistance patterns of the Gram-negative BSIs using the disk elution test

Table S9 | Known colistin (polymyxin E) resistance genes detected in the assembly of the Gram-negative BSIs based on RGI analysis

Table S10 | Catalog/Clone number for Flow Antibodies

Table S11 | Self-reported racial and ethnic categories by group

Fig. S1 | Actual oral vancomycin-polymyxin B administration.

Fig. S2 | Samples collected and sequenced per patient.

Fig. S3 | Number of reads per sample at each pre-processing step.

Fig. S4 | Taxonomy and Shannon diversity of all samples (Genus level)

Fig. S5 | Shannon diversity is similar between the GD and no-GD groups based on intention-to-treat analysis at the genus taxonomic level.

Fig. S6 | Change in Shannon diversity at 2 weeks relative to baseline sample

Fig. S7 | Shannon diversity with all samples in the study.

Fig. S8 | Principal coordinates analysis (PCoA) showing most samples are not different from one another (beta diversity).

Fig. S9 | No correlation between doses of vancomycin-polymyxin B received and Shannon diversity in GD arm

Fig. S10 | Kaplan-Meier curves of OS and PFS.

Fig. S11 | Relative abundance of gut microbes and the microbe causing the BSI in relation to antibiotic administration and Shannon diversity over time for patients C03 and C11.

Fig. S12 | Multiple BSI isolates are nearly identical or identical to those found in the gut microbiota prior to the BSI.

Fig. S13 | Antibiotic, antifungal, and antiviral administration timing

Fig. S14 | Potential model of oral vancomycin-polymyxin B on the microbiota.

Document S1 | Informal survey of pediatric HSCT centers
Document S2 | Clinical inclusion and exclusion criteria
Document S3 | CONSORT checklist
Document S4 | Relevance of the vancomycin polymyxin B gut decontamination strategy to a larger study
Supplementary References

Supplemental Table 1. Vancomycin and Polymyxin B administration details

Participants assigned to arm A received non-absorbable, oral vancomycin-polymyxin B capsules according to body surface area; approximately 250-500 mg/m2 BSA of vancomycin and approximately 125-250 mg/m2 of polymyxin B.
Each capsule contains 125 mg of vancomycin and 62.5 mg of polymyxin B.

Body Surface Area (m^2)	Dose of Vancomycin-Polymyxin B Capsules
< 0.5 m^2	1 cap PO TID
0.5 – 0.99 m^2	2 cap PO TID
1 – 1.49 m^2	3 cap PO TID
≥ 1.5 m^2	4 cap PO TID

Vancomycin-polymyxin B were given orally or via enteric feeding tubes. Doses were repeated if the participant vomited within 30 minutes of medication administration. For administration via enteric feeding tubes, vancomycin-polymyxin B capsules may be opened and the contents dissolved in 2-3 mL bottled or sterile water at room temperature. See Supplemental Figure 1 for actual administration during the study.

Supplemental Table 2. Bloodstream infections

Subject	Arm	Organism	Day of BSI episode relative to transplant
C03	B (no-GD)	*Leclercia adecarboxylata*	22
C04	B (no-GD)	*MRSA (Staphylococcus aureus)* *<br>*Klebsiella oxytoca*<br>*MRSA (Staphylococcus aureus)* *	5<br>18<br>94
C10	B (no-GD)	*Escherichia coli*	8
C11	A (GD)	*Bacillus* by clinical microbiology lab<br><br>Sequencing results of BSI isolate:<br>*Lysinibacillus fusiformis*<br>(also called *Bacillus fusiformis*)	31
C20	B (no-GD)	*Staphylococcus epidermidis* *	23
C22(B)	B (no-GD)	*Rothia dentocariosa*<br>*Enterococcus faecium*	6 #<br>20 #

* non-mucosal barrier injury (MBI) pathogens defined by the National Healthcare Safety Network (NHSN) criteria (Jan. 2021) of the Centers for Disease Control (CDC). Gut decontamination with vancomycin-polymyxin B (GD), methicillin-resistant *Staphylococcus aureus* (MRSA), bloodstream infection (BSI).

BSI occurred during the second transplant; numbering is according to the date relative to the start of the second transplant

Supplemental Table 3.

(A) Shannon diversity at the species level of patient samples at baseline (prior to any GD exposure) and two-weeks after transplant.

Species	GD (Baseline)	GD (2-week)	No-GD (Baseline)	No-GD (2-week)
Median	3.55	2.36	3.31	3.09
Range	2.1 - 4.5	0.03 - 5.3	1.2 - 4.2	2.1 - 3.7

(B) Shannon diversity at the genus level of patient samples at baseline (prior to any GD exposure) and two-weeks after transplant.

Genus	GD (Baseline)	GD (2-week)	No-GD (Baseline)	No-GD (2-week)
Median	2.23	1.79	1.47	1.65
Range	0.6 - 3.0	0.05 - 4.5	0.3 - 3.0	1.2 - 2.4

Supplemental Table 4. Shannon diversity baseline and 2-week comparisons. p-value for comparing baseline samples prior to GD exposure and 2-week timepoints. Wilcoxon sign-rank for matched samples, Wilcoxon rank-sum test (Mann-Whitney *U* test) for unrelated (GD vs No-GD) comparisons

Cohort	Comparison	*p*-value for Shannon diversity (Genus)	*p*-value for Shannon diversity (Species)
GD	Baseline vs 2-weeks post HCT	>0.99	0.106
No-GD	Baseline vs 2-weeks post HCT	0.922	0.492
Overall, at Baseline	GD vs No-GD	0.315	0.353
Overall, at 2-weeks post HCT	GD vs No-GD	0.796	0.436

Supplemental Table 5. Duration of antibiotic exposure within the first 30 days post-HCT

	# Days of antibiotics [median (range)]		p-value
	GD (n=10)	no-GD (n=10)	
CLASSES			
Cephalosporins	0 (0, 11)	0 (0, 8)	0.80
Fluoroquinolones	2.5 (0, 24)	4 (0, 17)	0.40
Broad spectrum with anaerobic coverage (Amp/Sulbactam, Pip/Tazo, Meropenem)	13 (0, 39)	17 (0, 32)	0.68
INDIVIDUAL ANTIBIOTICS			
Vancomycin (IV)	0 (0, 22)	2 (0, 19)	0.77
Meropenem	0 (0, 19)	0 (0, 24)	0.69
Piperacillin/Tazobactam	7 (0, 31)	11.5 (0, 26)	0.49
Trimethoprim-sulfamethoxazole **	5 (4, 5)	5 (0, 5)	0.23

** Prophylactic dose Trimethoprim / Sulfamethoxazole (TMP/SMX; cotrimoxazole) analyzed from day -5 to day +30; three patients (C04, C12 and C22) in the no-GD arm had pentamidine during the pre-treatment phase (and thus no TMP/SMX during this window). Subject C04 had prophylactic dose TMP/SMX from day 25 to 27.

Supplemental Table 6. Antibiotic susceptibility testing of blood culture isolates.

Subject	BSI Organism	AMP	Oxacillin	Pip/Tazo	Amp/Sulbactam	Cefazolin (1st)	CFTX (3rd)	Cefotax. (3rd)	Ceftaz. (3rd)	Cefepime (4th)	Clindamycin	Amikacin	Gentamicin	Erythromycin	Meropenem	Ciprofloxacin	TMP/SMX	Tetracycline	Vancomycin	Linezolid	synercid (quinupristin/dalfopristin)	Tigecycline	Metronidazole	Colistin
C03	*Leclercia adecarboxylata*	S		S	S		S		S	S		S	S		S	S	S							I
C04	*MRSA* (first infection, day +5)		R		R	R	R	R			R			R			S	S	S	S	S	S		
C04	*MRSA* (second infection, day +94)		R		R	R	R	R			R			R			S	S	S	S	S	S		
C04	*Klebsiella oxytoca_1*			R	R		I			S		S	S		S	S	S							R
C04	*Klebsiella oxytoca_2*				R		S			S		S	S		S	S	S							R
C10	*Escherichia coli_1*	R		R	R		R		R	R		S	S		S	R	R							I
C10	*Escherichia coli_2*	R		R	R		R		R	R		S	S		S	R	R							I
C11	*Bacillus* (not anthracis, not cereus)		S				I						S		S				S					
C20	*Staph epidermidis (CoNS)*		R		R	R	R	R			R			R			R	S	S	S	S	S		
C22	*Rothia dentocariosa* (beta-lactamase negative)		S	S	S										S								I	
C22	*Enterococcus faecium*	R	R																S			S		

S, susceptible; I, intermediate; R, resistant.
_1 (strain #1) _2 (strain #2) on the same day; vs (first) separate time infections (second)
Red = Gut Decontamination (GD). Blue = no-GD

A total of 9 bloodstream infection (BSI) events occurred in 6 patients (also see Figure 6 and Supplemental Figure 11). 11 clinical isolates were obtained from the clinical microbiology lab. Listed are the antibiotics along with the individual antibiotic sensitivity from the clinical microbiology laboratory of all isolates; S = susceptible, I = intermediate, R = resistant. For strains isolated on the same day, an underscore is noted (e.g., _1 = strain 1, _2 = strain 2). See Supplementary Table 8 for colistin (polymyxin E) interpretation.

Antibiotic susceptibility testing on isolates from bloodstream infections was performed by the Clinical Microbiology Laboratory at Boston Children's / Dana Farber Cancer Center, except for colistin (polymyxin E), which was performed at Stanford Health Care Clinical Microbiology Laboratory using the disk elution test as previously described (1). Minimal inhibitory concentrations (MIC) for Enterobacteriales were interpreted using breakpoints according to Clinical and Laboratory Standards Institute (CLSI) (2) and the European Committee on Antimicrobial Susceptibility Testing (EUCAST) (3).
AMP = Ampicillin, PipTazo = Piperacillin / Tazobactam, Amp/Sulbactam = Ampicillin / Sulbactam, CFTX (3rd) = Ceftriaxone (third-generation cephalosporin), Cefotax. (3rd) = Cefotaxime (third-generation cephalosporin), Ceftaz. (3rd) = Ceftazidime (third-generation cephalosporin), Clinda = Clindamycin, Mero = Meropenem, Cipro = Ciprofloxacin, TMP/SMX = Trimethoprim / Sulfamethoxazole (cotrimoxazole), Vanc = Vancomycin

Supplemental Table 7. BSI Reads and Coverage based on estimated Genome size

Sample	Raw reads	Deduplicated Reads	Trimmed Reads	Reads after host removal	Estimated size of bacterial genome (Mb)	Coverage
Leclercia adecarboxylata	31,620,619	21,161,570	18,403,419	17,355,375	4.62	563x
MRSA (first infection, day +5)	31,300,420	17,730,130	14,543,633	9,929,725	2.83	526x
MRSA (2nd infection, day +94)	26,421,425	15,932,677	13,362,581	9,332,184	2.83	495x
Klebsiella oxytoca (Strain #1)	43,652,689	28,556,138	24,944,940	23,524,208	6.02	586x
Klebsiella oxytoca (Strain #2)	27,575,433	19,262,474	16,824,826	15,779,072	6.02	393x
E. coli (Strain #1)	46,579,261	28,337,882	24,539,704	22,746,871	5.12	666x
E. coli (Strain #1)	40,899,722	23,939,870	20,715,391	19,224,972	5.12	563x
Bacillus *(Lysinibacillus fusiformis / Bacillus fusiformis)*	33,318,357	20,722,144	17,793,904	13,650,888	5.06	405x
*Staphylococcus epidermidis**	45,968,718	25,508,418	22,100,194	20,450,659	2.52	N/A*
Enterococcus faecium	26,366,144	14,524,716	11,253,613	7,532,822	2.92	387x
Rothia dentocariosa	30,518,763	17,751,972	15,060,078	11,092,660	2.49	668x
Mean	34,929,232	21,220,726	18,140,208	15,510,858	Mean	525x
Median	32,469,488	20,941,857	17,967,056	15,644,965	Median	545x

* We were unable to do strain-level analysis as the original *Staph. epidermidis* BSI-causing isolate was archived, but upon sequencing was identified to be *E. coli*, a likely contaminant of the archived culture.

Coverage is the number of times a genome has been sequenced. Based on the Lander-Waterman equation (4) of $C = LN / G$

C = Coverage
L = read length
N = number of reads
G = haploid genome length

This is estimated by the number of reads after host removal over the estimated genome size. In general, we require 5-10x coverage for taxonomic identification, and 30-100x coverage for assembly of a genome (though coverage may need to be greater for highly repetitive regions, which may not be resolved with short-read sequencing).

Supplemental Table 8. Colistin (polymyxin E) resistance patterns of the Gram-negative BSIs using the disk elution test as previously described (1).

Gram-negative BSI organism	MIC	Interpretation
Leclercia adecarboxylata	<1 ug/mL	Intermediate
Klebsiella oxytoca (Strain #1)	>4 ug/mL	Resistant
Klebsiella oxytoca (Strain #2)	>4 ug/mL	Resistant
E. coli (Strain #1)	<1 ug/mL	Intermediate
E. coli (Strain #2)	<1 ug/mL	Intermediate

Minimal inhibitory concentrations (MIC) were interpreted using breakpoints according to Clinical and Laboratory Standards Institute (CLSI) (2) and the European Committee on Antimicrobial Susceptibility Testing (EUCAST) (3); Epidemiological cut-offs are approximately 2 ug/mL for both Klebsiella and E. coli based on EUCAST, and extrapolated to *Leclercia* based on Enterobacterales susceptibility.

Supplemental Table 9. Known colistin (polymyxin E) resistance genes detected in the assembly of the Gram-negative BSIs based on RGI analysis (5) (strict and perfect hits only).

Antibiotic Resistance Gene:	*Leclercia*	*Klebsiella*_#1	*Klebsiella* _#2	*E. coli*_#1	*E. coli*_#2
PmrF	-	+	+	+	+
PmrA	-	-	-	-	-
arnA	-	-	-	-	-
arnT	-	+	+	-	-
eptA	-	-	-	+	+
eptB	-	-	-	+	+
acrA	-	-	-	+	+
acrB	+	+	+	+	+
acrR	+	-	-	+	+
kpnEF	+	+	+	+	+
MCR-9.1	+	-	-	-	-

+ = Gene detected in assembled genome

- = Gene not detected

Supplemental Table 10. Catalog/Clone number for Flow Antibodies
Conjugated monoclonal antibodies used in flow cytometry analysis. Gating strategy has previously been described in Alho, et al., Blood (2016) (6).

**Surface Antigens**
CD3 - BV450 (clone UCHT1; BDBioscience)
CD4 - Apc-Cy7 (clone RPA-T4; BDBioscience)
CD8 - BV510 (clone RPA-T8; BioLegend)
CD19 - APC (clone HIB19; BD Pharmingen)
CD25 - PE-Cy7 (clone M-A251; BD Pharmingen)
CD27 - PE-Cy5, eBioscience, clone 0323
CD45RA - PeCy7 (clone HI100; BDBioscience)
CD45RO - FITC (clone UCHL1; BD Pharmingen)
CD56 - PE (clone B159; BD Pharmingen)
CD62L - APC (clone DREG-56; BDBioscience)
CD127 - PE-Cy5 (clone eBioRDR5; eBioscience)

Supplemental Table 11. Self-reported racial and ethnic categories by group

Per Office of Management and Budget (OMB) Directive 15, Revisions to the Standards for the Classification of Federal Data on Race and Ethnicity (https://grants.nih.gov/grants/guide/notice-files/not-od-15-089.html), the following are the self-reported racial and ethnic groups for all 20 subjects

	GD (N=10)		No GD (N=10)		All (N=20)	
	N	%	N	%	N	%
**American Indian or Alaska Native**	--	--	--	--	--	--
**Asian**	--	--	--	--	--	--
**Black or African American**	--	--	--	--	--	--
**Hispanic or Latino**	4	40	2	20	6	30
**Native Hawaiian or Other Pacific Islander**	--	--	--	--	--	--
**White**	5	50	7	70	12	60
**Other or declined to state**	1	10	1	10	2	10

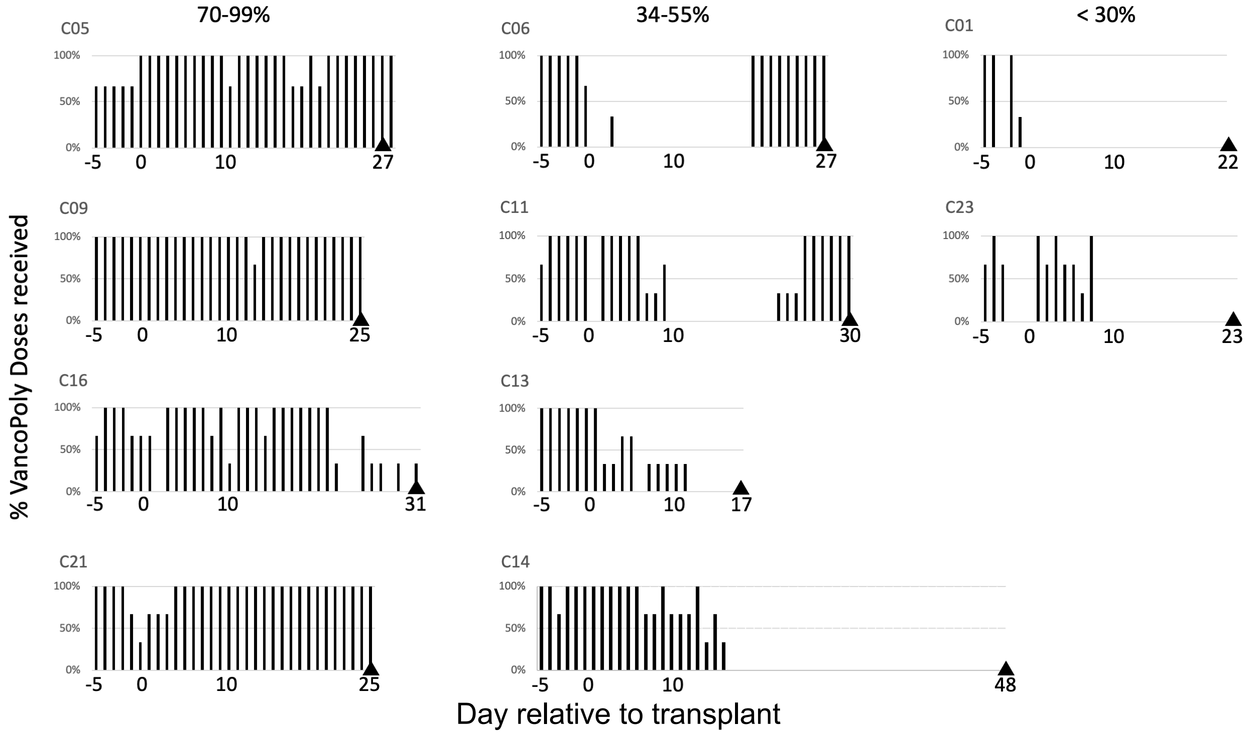

Supplemental Fig. 1 | Actual oral vancomycin-polymyxin B administration. Percent of doses taken daily for all 10 patients receiving gut decontamination (arm A) with oral Vancomycin-Polymyxin B starting from transplant day -5 through neutrophil engraftment (triangle) for each patient. Patients are grouped by 70-99% of doses taken (left), 34-55% (center), and <33% (right). Median day of neutrophil engraftment is day +25 for all patients, +26 for arm A only.

A.

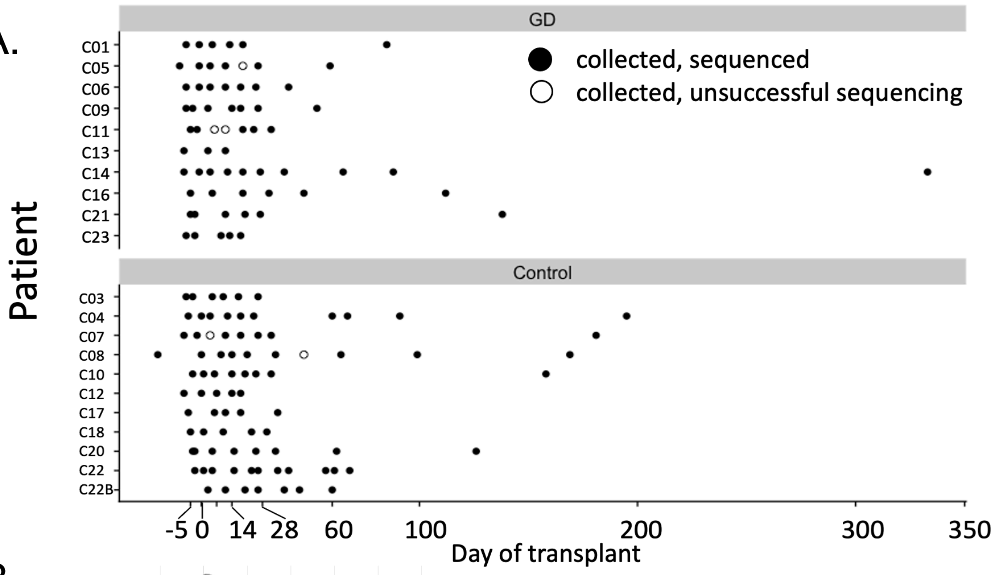

B.

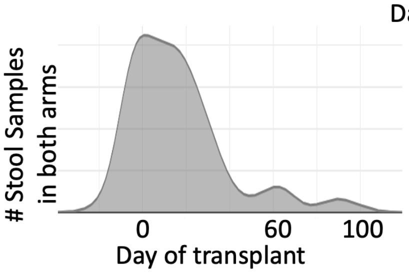

C.

Mean, median, and range of samples collected per patient

	GD	No-GD	TOTAL
**Mean**	**6.4**	**8.3**	**7.4**
**Median**	**6.5**	**8**	**7**
**Range**	**3 to 10**	**5 to 18**	**3 to 18**

Supplemental Fig. 2 | Samples collected and sequenced per patient.
(A) Stool samples collected per patient in relation to days relative to transplantation, divided into gut decontamination (GD with vancomycin-polymyxin B) and no-GD.
Closed circles are stool samples able to be sequenced, and open circles are stool samples collected but unable to be sequenced due to limited biomass. Patient 22 did not engraft and received two transplants, divided into C22 and C22B. 8 samples are greater than day 100.
(B) Total number of stool samples across all patients are focused from day -5 to the first 30 days.
(C) Table showing numbers of samples collected per patient. Total number sequenced: 142 /147 = 97% of collected samples.
18 samples total for patient 22, (divided into 1st and 2nd transplant, with 11 samples and 7 samples, respectively), and 3 samples from healthy siblings (not included in this table; Data from healthy siblings included in Supplemental Figures 4 and 8).

Mean	Median
63.7×10^6	72.3×10^6
41.4×10^6	45.4×10^6
35.2×10^6	38.2×10^6
16.6×10^6	16.9×10^6

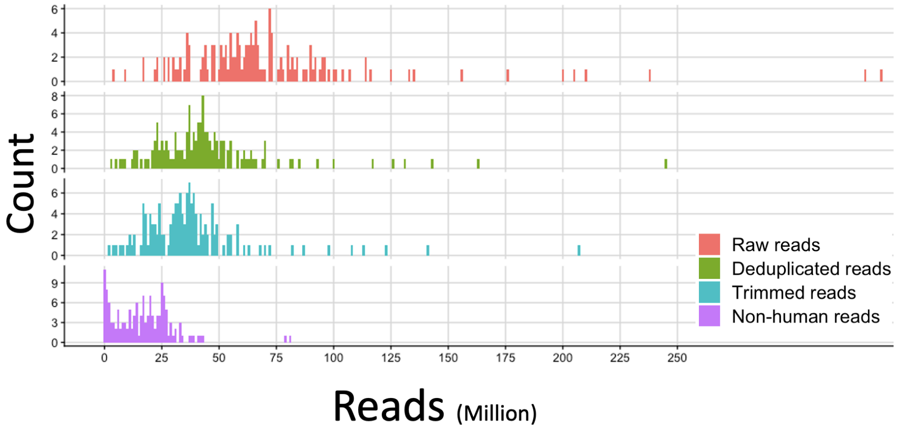

Supplemental Fig. 3 | Number of reads per sample at each pre-processing step.
Raw reads were demultiplexed by unique barcodes (bcl2fastq v2.20.0.422, Illumina).
Reads were deduplicated to remove PCR artifacts and any duplicates with SuperDeduper v1.4.
Deduplicated reads were trimmed using TrimGalore v0.4.4.
Finally, any human reads aligning to human genome (hg19.fa) were removed prior to analysis.
Two samples were mostly human reads and had <100,000 microbial reads after removing human reads, however these samples had 21.3 and 48.8 x 10^6 reads after deduplication and trimming.
The final mean read-depth was 16.6 Million reads per sample.

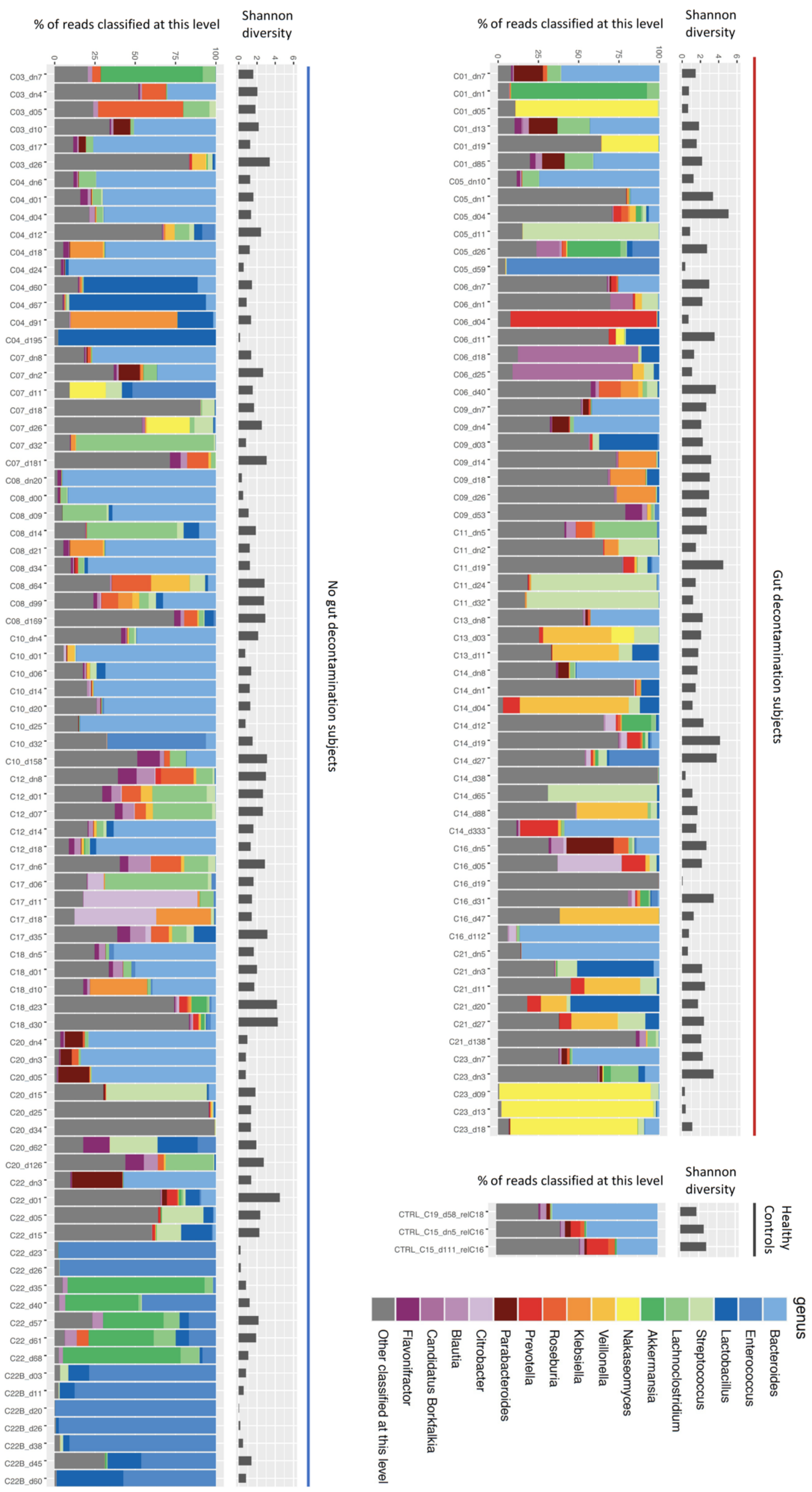

Supplemental Fig. 4 | Taxonomy of all samples displayed as the relative abundance as a percentage of classified reads at the genus level. Shannon diversity at each time point is shown as a bar. Subject sample is coded as follows: Subject ID_day of sample (e.g., dn7 = day -7; d01 = day +1).

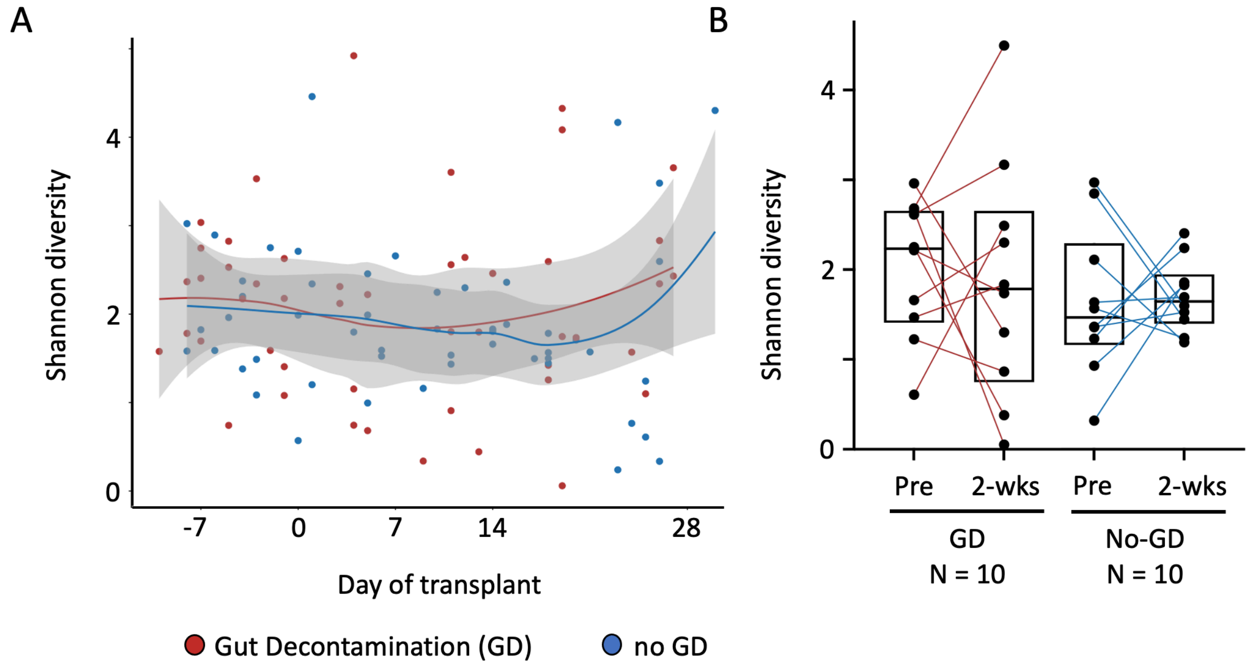

Supplemental Figure 5 | Shannon diversity is similar between the GD and no-GD groups based on intention-to-treat analysis at the genus taxonomic level. Red are samples from patients undergoing GD and blue is no-GD arm.

(A) Shannon diversity over time analyzed at the genus level using local polynomial regression fitting (LOESS-locally estimated scatterplot smoothing of the mean Shannon diversity) showing similarity between the two groups. N=48 samples from 10 patients in GD arm, N= 51 samples from 10 patients in no-GD arm. **(B)** Shannon diversity of individual patient data from pre-transplant (before GD antibiotics) to 2 weeks post HCT connected with a line. Boxes shown are the median with hinges at the 25% and 75%. All comparisons not significant (see Supplemental Table 4) using Wilcoxon rank-sum test. N=10 subjects GD arm, N=10 subjects no-GD arm. Comparison at the species level (see Figure 3 and Supplemental Table 4) is also not significant.

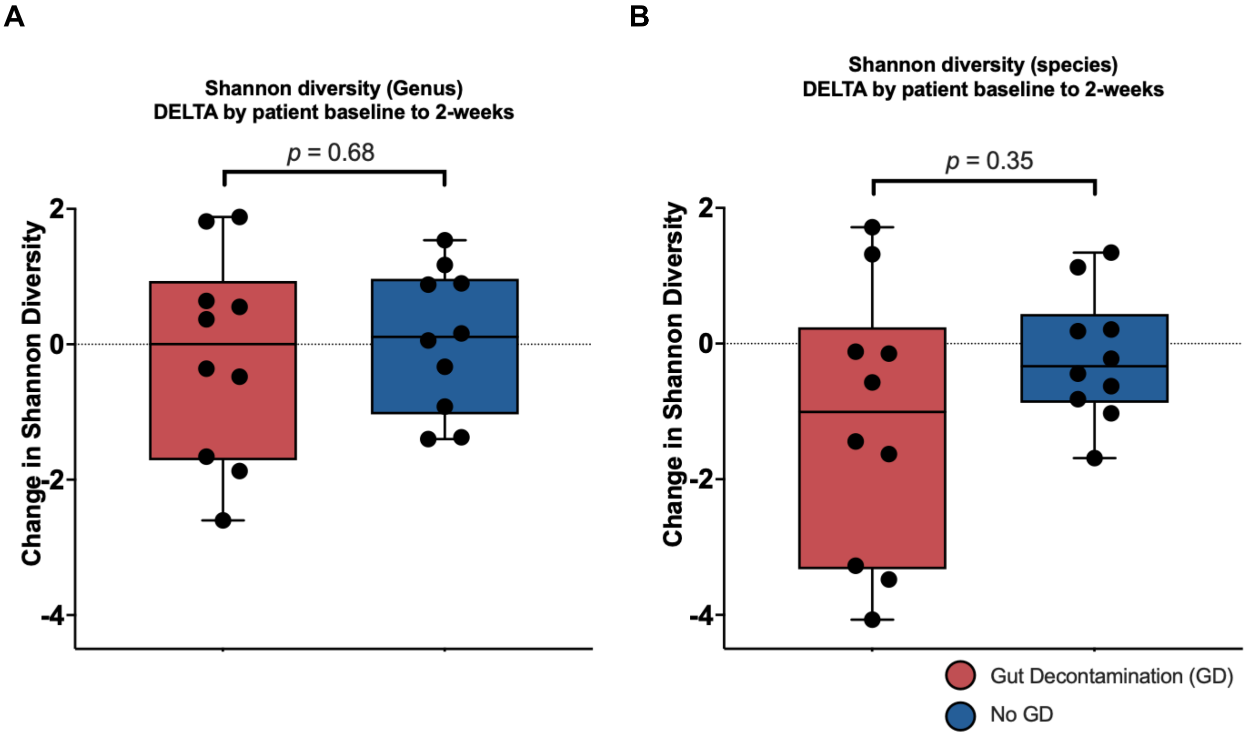

Supplemental Figure 6 | Change in Shannon diversity at 2 weeks relative to baseline sample. Box and whiskers plot of the median change Shannon diversity index at 2 weeks relative to the baseline samples for each individual subject at the **(A)** Genus and **(B)** species level. Lower and upper hinges correspond to the first and third quartiles (the 25th and 75th percentiles). Boxes are labeled as gut decontamination (red) and no-GD (blue). Whisker extends from the hinge to the maximum or minimum value. Statistical calculations are not significant using Wilcoxon rank-sum test (Mann-Whitney *U* test).

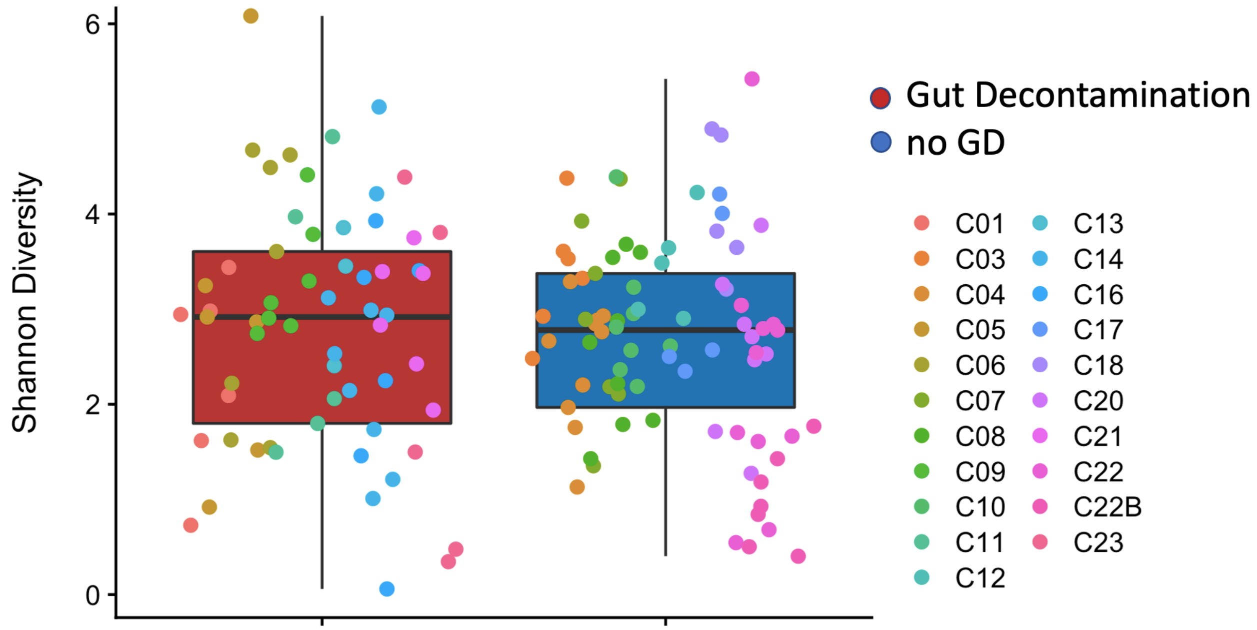

Supplemental Fig. 7 | Shannon diversity with all samples in the study.
Box and whiskers plot of the mean Shannon diversity index shown at the species level with the lower and upper hinges correspond to the first and third quartiles (the 25th and 75th percentiles). Boxes are labeled as gut decontamination (red) and no-GD (blue).
Whisker extends from the hinge to the largest value no further than 1.5 * inter-quartile range (IQR) from the hinge. Individual patient samples are colored dots according to the legend.
Statistical test via Wilcoxon rank-sum test with FDR, $p_{adj.} = 0.51$. Subject C22 divided into C22 (first transplant) and C22B (second transplant) for clarity of diversity trends between transplants.

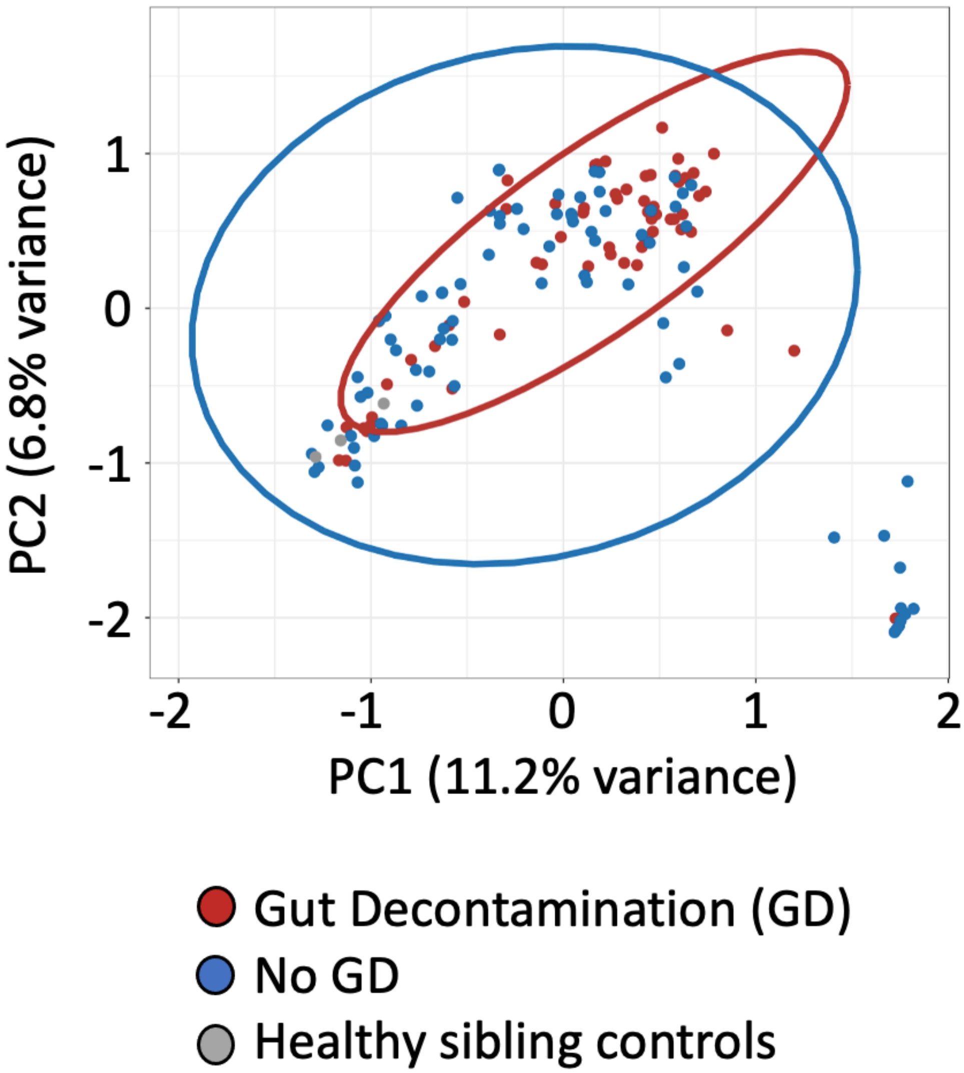

Supplemental Figure 8 | Principal coordinates analysis (PCoA) showing most samples are not different from one another (beta diversity).
Samples from patients undergoing GD (red), no-GD (blue), and three samples from two healthy sibling controls (gray).
PCoA using a Bray-Curtis dissimilarity index at species level indicates that 11.2% and 6.8% of the variance can be explained by the first two principal coordinates. Ellipses represent the 95% CI for the GD and no-GD groups. A group of outliers (from 3 different patients, C05 (GD), C07 (no-GD), and C22 (no-GD)) in the lower right corner include any sample with >45% relative abundance of *Enterococcus faecium.*

As a control, stool samples from two healthy sibling donors (gray circles) were also collected to serve as a comparison to the HCT patients, and were similar at the time points collected (in the lower left corner). Between Control Sibling C15 and Patient C16 (GD arm), the Analysis of Similarity (ANOSIM) statistic R was -0.3125 with a significance of 0.928 after 999 permutations. Between Control Sibling C19 and Patient C18 (no-GD arm) the ANOSIM statistic R was -0.04 with a significance of 0.5 after 999 permutations.

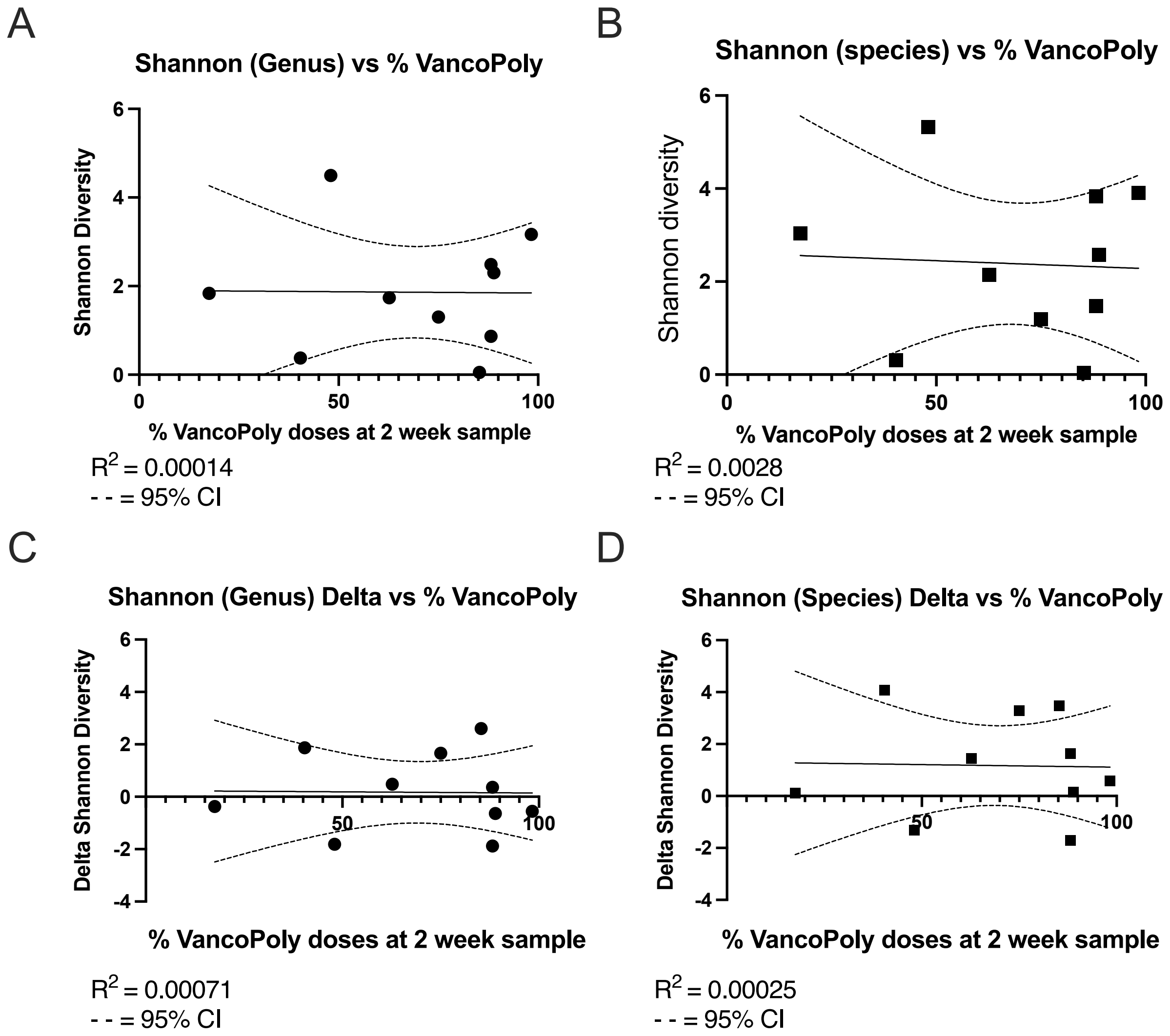

Supplemental Fig. 9 | No correlation between doses of vancomycin-polymyxin B received in the GD arm and Shannon diversity.

Shannon diversity at 2-week sample compared to % of vancomycin-polymyxin B doses received at the (A) genus and (B) species taxonomic levels. Change in Shannon Diversity at 2-weeks (2-week over Baseline) compared to % of vancomycin-polymyxin B doses received at the (C) genus and (D) species taxonomic levels. Curve fit with simple linear regression and R2 value listed below each graph. Dashed line is the 95% CI for the curve.

Closed circles (A) and (C) are change in diversity at the genus taxonomic level, and closed squares (B) and (D) are change in diversity at the species taxonomic level.

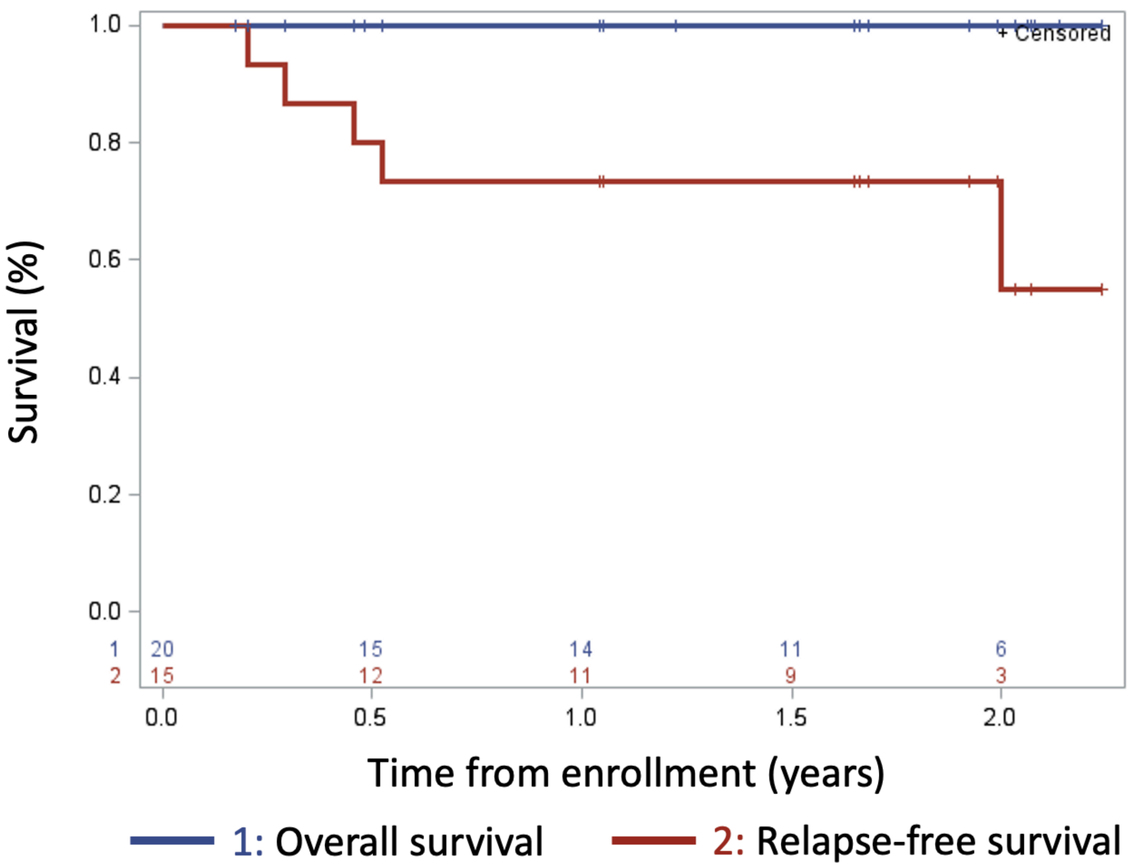

Supplemental Figure 10 | Kaplan-Meier curves of OS (n=20 overall) and relapse-free survival (n=15 with malignancy)

For OS at 1-year, no patients died; the 1-year OS was 100% (n=20). For relapse-free survival, of the 15 patients with a malignancy, five patients had a relapse; the 1-year relapse-free survival was 73±11.4% (n=15).

A.

C03

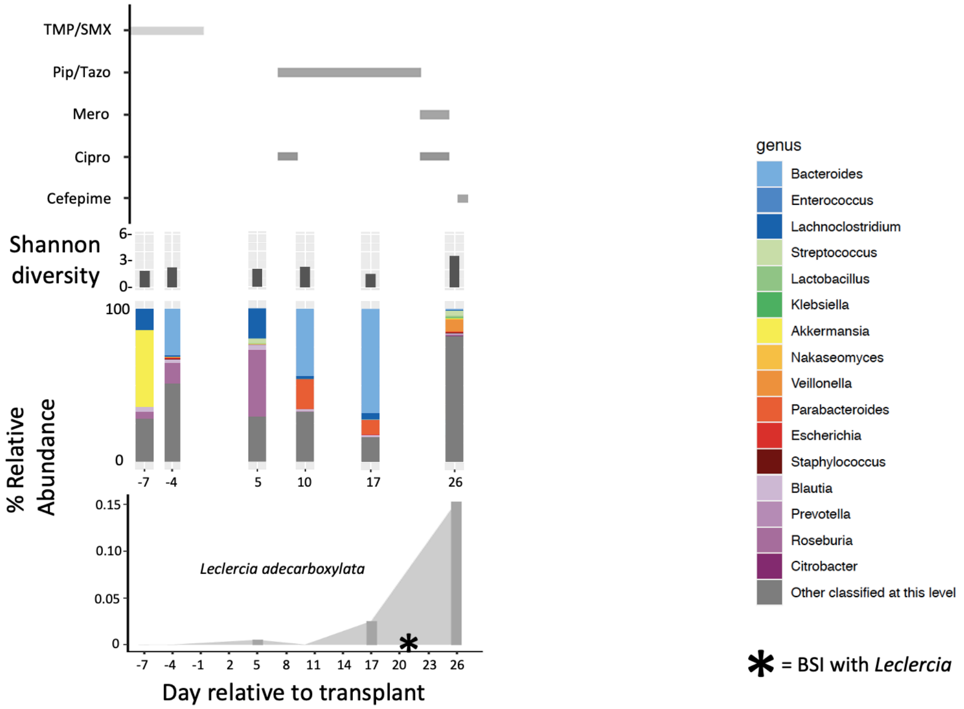

Supplemental Fig. 11 | Relative abundance of gut microbes and the microbe causing the BSI in relation to antibiotic administration and Shannon diversity over time for patients C03 and C11.

(A). Results for patient C03
Days relative to the course of the transplant on the X-axis, (from top to bottom on the Y-axis) antibiotic administration, alpha (Shannon) diversity, relative abundance of microbes in the stool samples at the genus taxonomic level (with organisms listed by color according to the key at the right), and relative abundance of in the gut of the BSI-causing organism with the date of the BSI shown as an asterisk (*).

B.

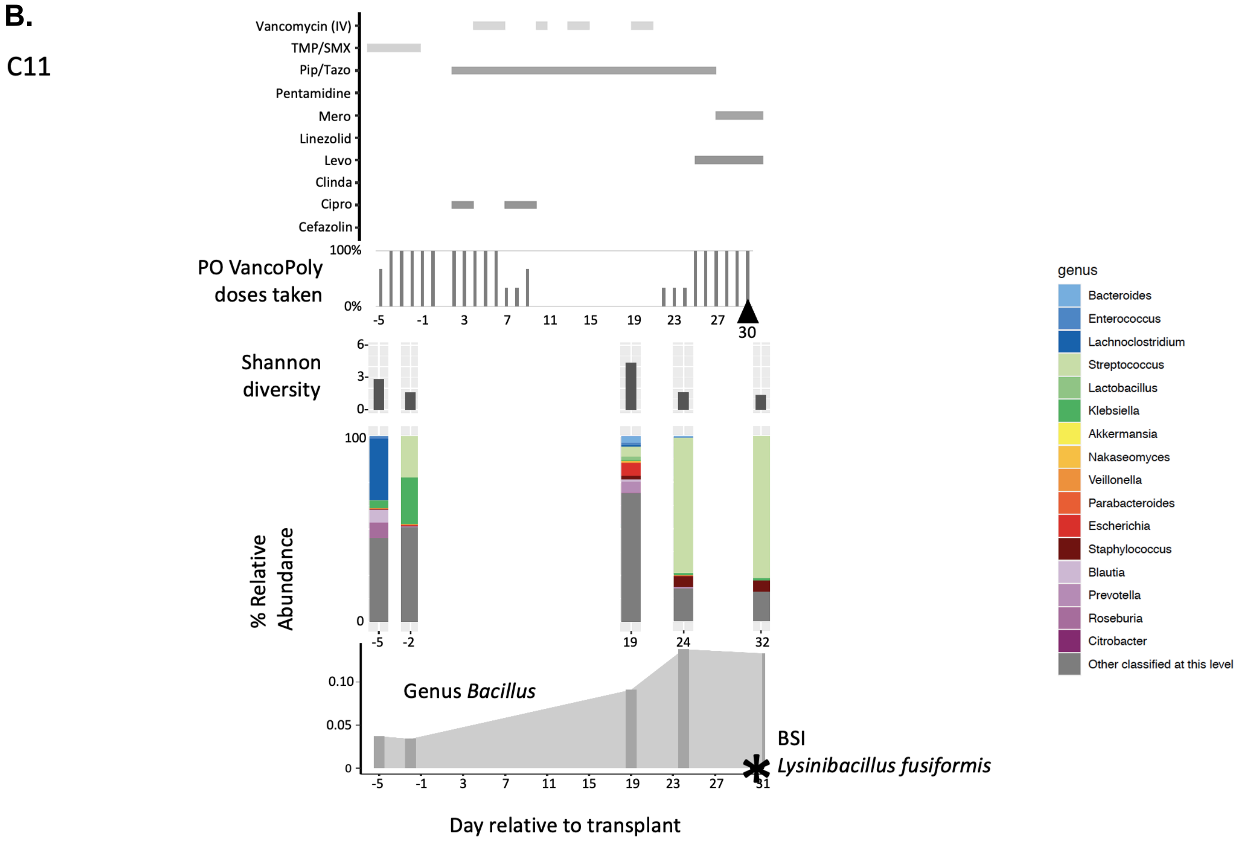

(B). Results for patient C11
Days relative to the course of the transplant on the X-axis, (from top to bottom on the Y-axis) antibiotic administration, percentage of the oral (PO) vancomycin-polymyxin b doses taken from day -5 through engraftment, alpha (Shannon) diversity, relative abundance of microbes in the stool samples at the genus taxonomic level (with organisms listed by color according to the key at the right), and relative abundance of Bacillus genus over time. Note that the BSI causing organism, *Lysinibacillus fusiformis*, was not found in any of the stool samples for this patient suggesting the BSI did not originate from the gut. Date of *Lysinibacillus fusiformis* BSI shown as an asterisk (*).

Cipro = Ciprofloxacin, Clinda = Clindamycin, Levo = Levofloxacin, Mero = Meropenem, PipTazo = Piperacillin / Tazobactam, TMP/SMX = Trimethoprim / Sulfamethoxazole (cotrimoxazole)

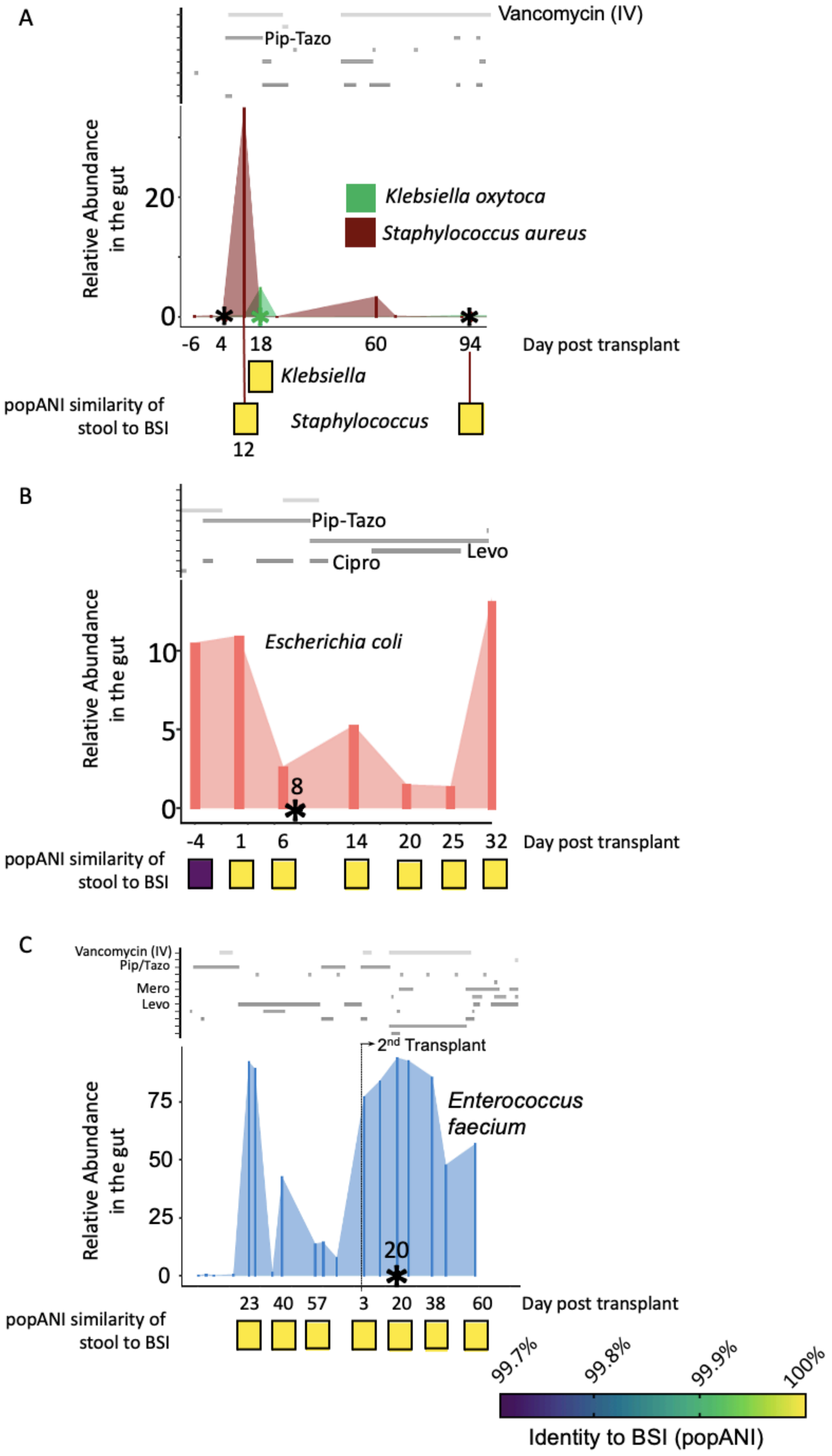
A
Vancomycin (IV)
Pip-Tazo
Relative Abundance in the gut
20
0
Klebsiella oxytoca
Staphylococcus aureus
-6 4 18 60 94
Day post transplant
Klebsiella
popANI similarity of stool to BSI
Staphylococcus
12
B
Pip-Tazo
Levo
Cipro
Relative Abundance in the gut
10
5
0
Escherichia coli
8
-4 1 6 14 20 25 32
Day post transplant
popANI similarity of stool to BSI
C
Vancomycin (IV)
Pip/Tazo
Mero
Levo
2nd Transplant
Relative Abundance in the gut
75
50
25
0
Enterococcus faecium
20
23 40 57 3 20 38 60
Day post transplant
popANI similarity of stool to BSI
99.7%
99.8%
99.9%
100%
Identity to BSI (popANI)

Supplemental Fig. 12 | Multiple BSI isolates are nearly identical or identical to those found in the gut microbiota prior to the BSI.
5 BSIs from 3 patients had sufficient coverage to be evaluated by *inStrain*. Relative abundance of the species in the gut that corresponds to the BSI. Above is the administration of antimicrobials, with select antibiotics labeled. Below is the identity of the stool sample to the BSI measures as a population average nucleotide identity (popANI) at the time relative to the transplant date. The relative scale of the popANI is shown in the lower right corner.

A. *Klebsiella* BSI on day +18 has 100% popANI compared to the BSI on the same day. *MRSA* BSI on days +5 and +94. The stool sample from day +12 had *S. aureus* at 35% relative abundance that was identical to the BSI on day +5 (100% popANI, zero SNPs detected in 2.8 Mb of sequence compared) and nearly identical (99.9999% popANI) to BSI on day +94.

B. *E. coli* BSI on day +8 is identical (100% popANI, 5 Mb compared) to the six stool samples collected from days +1 to +32. A distinct strain was present in the stool at day -4 (99.7% popANI to BSI and other stool samples).

C. *E. faecium* BSI on day +20. *E. faecium* stool samples from day +23 of the first transplant through the end of the study (14 samples total) are nearly identical (>99.9999% popANI) to the BSI strain on day +20

See Table 3 for concordance values (coverage, popANI, population SNPs, and conANI) of BSI isolate to stool metagenomes.

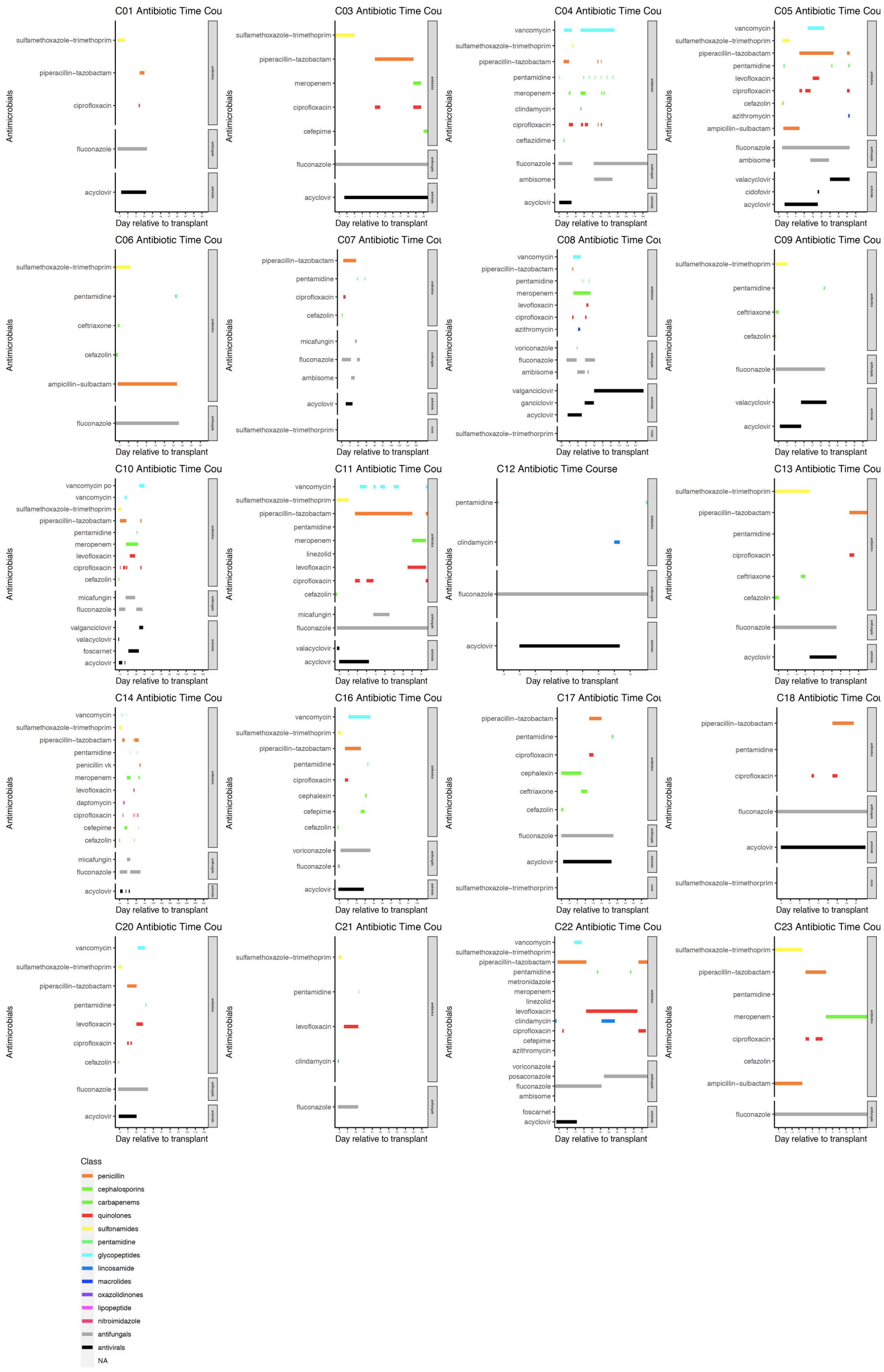
C01 Antibiotic Time Cou
C03 Antibiotic Time Cou
C04 Antibiotic Time Cou
C05 Antibiotic Time Cou
C06 Antibiotic Time Cou
C07 Antibiotic Time Cou
C08 Antibiotic Time Cou
C09 Antibiotic Time Cou
C10 Antibiotic Time Cou
C11 Antibiotic Time Cou
C12 Antibiotic Time Course
C13 Antibiotic Time Cou
C14 Antibiotic Time Cou
C16 Antibiotic Time Cou
C17 Antibiotic Time Cou
C18 Antibiotic Time Cou
C20 Antibiotic Time Cou
C21 Antibiotic Time Cou
C22 Antibiotic Time Cou
C23 Antibiotic Time Cou
Antimicrobials
Day relative to transplant
Class
penicillin
cephalosporins
carbapenems
quinolones
sulfonamides
pentamidine
glycopeptides
lincosamide
macrolides
oxazolidinones
lipopeptide
nitroimidazole
antifungals
antivirals
NA

Supplemental Fig. 13 | Antibiotic, antifungal, and antiviral administration timing. For antibacterial agents, systemic antibacterial prophylaxis included:

- TMP/SMX for PJP/PCP pneumonia prevention: started on admission and continued until day -1,
- Ampicillin/Sulbactam for empiric coverage of oral flora in patients with poor dentition: started on admission and was continued through neutrophil engraftment or when replaced by another antibiotic with similar antimicrobial coverage, (e.g., at the time of first neutropenic fever),
- Single dose of IV cephalexin, cefazolin, or clindamycin was given in the operating room with central venous line (CVL) placement.

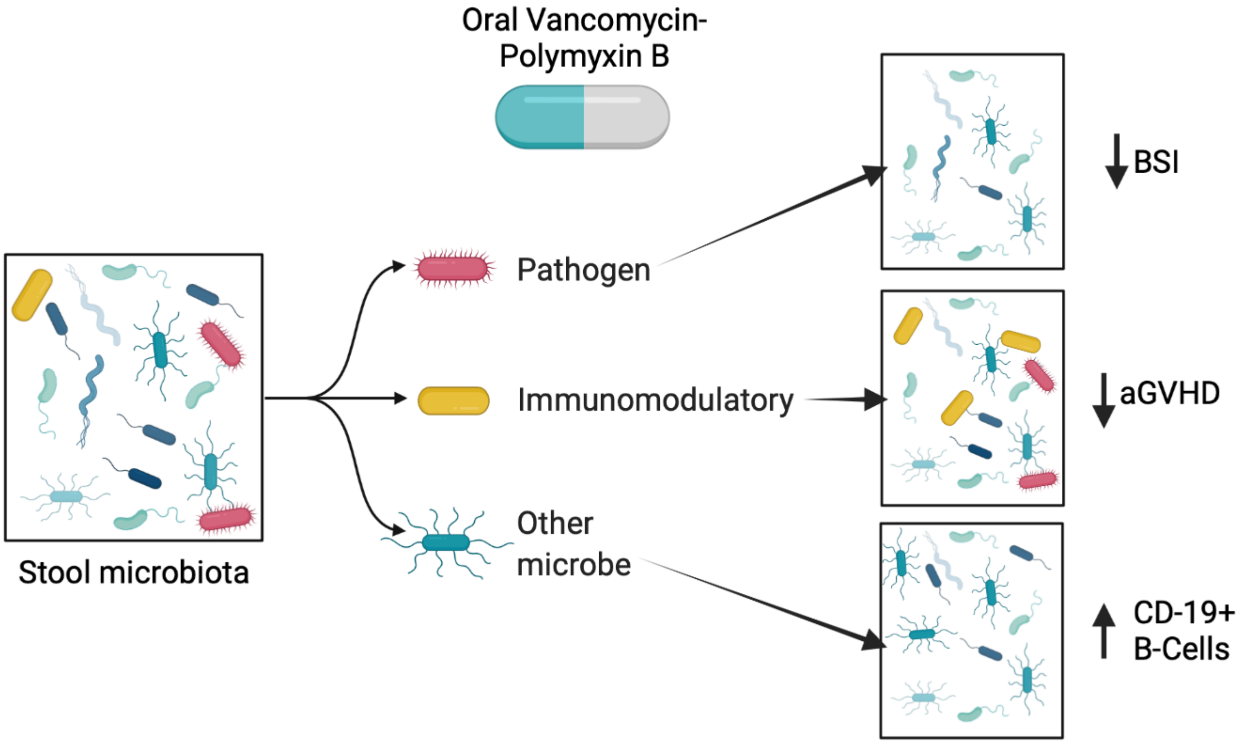

Supplemental Fig. 14 | Potential model of oral vancomycin-polymyxin B on the microbiota. Possible scenarios for presumed beneficial effects of oral vancomycin-polymyxin B gut decontamination on the microbiota. GD may decrease the number of pathogens in the gut that cause a BSI, may allow immunomodulatory bacteria to increase in number or create an environment where the immune system is less prone to the inflammatory aGVHD state, or allow expansion of other microbes that alter the host-microbiota interaction (such as changes in the number of CD-19+ B-cells).

Supplemental document 1 | Informal survey of pediatric HCT centers

Q1: Approximately how many pediatric allogeneic hematopoietic stem cell transplants does your center perform on an annual basis?

Prompt	Count	Pct
Less than 10 per year	6	19.35%
**10-25 per year**	**12**	**38.71%**
26-35 per year	4	12.90%
36-50 per year	6	19.35%
Greater than 50 per year	3	9.68%
**Aggregates**		
Count		31

Q2: What is your estimated incidence of acute graft-versus-host disease (GVHD) among pediatric patients at your center for recipients of MATCHED SIBLING donor stem cell transplants?

Prompt	Count	Pct
Less than 5%	4	13.33%
5-10%	8	26.67%
11-15%	7	23.33%
**16-20%**	**9**	**30.00%**
Great than 20%	2	6.67%
**Aggregates**		
Count		30

Q3: What is your estimated incidence of acute graft-versus-host disease (GVHD) among pediatric patients at your center for recipients of UNRELATED donor stem cell transplants?

Prompt	Count	Pct
Less than 5%	2	6.45%
5-10%	2	6.45%
11-15%	3	9.68%
16-20%	9	29.03%
**Great than 20%**	**15**	**48.39%**
**Aggregates**		
Count		31

Q4: Does your stem cell transplant program practice gut decontamination for acute GVHD prophylaxis?

Prompt	Count	Pct
Yes	3	9.38%
**No**	**29**	**90.63%**

Aggregates	
Count	32

Supplemental document 2 | Clinical inclusion and exclusion criteria

Inclusion Criteria:

- Eligibility Criteria for Patients Undergoing Allogeneic HSCT
  - Recipient of 9/10 or 10/10 (HLA-A, -B, -C, -DRB1, -DQB1) matched bone marrow allogeneic hematopoietic stem cell transplantation (HSCT) OR 4/6, 5/6 and 6/6 (HLA-A, -B, -DR) matched cord blood allogeneic HSCT.
  - Participants may have underlying malignant or non-malignant hematologic disease, except for primary immunodeficiency, as the indication for their allogeneic HSCT. Patients with immune dysregulation such as familial or secondary hemophagocytic lymphohistiocytosis (HLH) are eligible.
  - Participants may receive either a myeloablative or non-myeloablative(reduced-intensity) conditioning regimen. Anti-thymocyte globulin (ATG) in the conditioning regimen is permitted.
  - Graft-versus-host disease (GVHD) prophylaxis with any of the following agents: calcineurin inhibitor, and short-course methotrexate, with or without steroids, mycophenolate mofetil, and sirolimus.
  - Age ≥ 4 years old and toilet-trained. Participants must be able to deposit stool samples directly into stool collection containers. Stool specimens from diapers are difficult to obtain and are prone to more sampling error, particularly for loose or liquid stools which are common in the peri-transplant period.
  - Lansky/Karnofsky performance status ≥60%
  - Ability to understand and/or the willingness of their parent or legally authorized representative to sign a written informed consent document
- Eligibility Criteria for Healthy Bone Marrow Donors
  - Healthy individuals, ages ≥ 4 years and toilet-trained, who have been identified by BCH or DFCI providers as 9/10 or 10/10 (HLA-A, -B, -C, -DRB1, -DQB1 matched bone marrow donors for transplantation will also be eligible to participate in this study.

Exclusion Criteria:

- Patients undergoing allogeneic HSCT for correction of a primary immunodeficiency disorder (e.g. SCID).
- Patients with age ≤ 10 years undergoing HSCT with a matched sibling donor. These patients are at very low risk of acute GVHD and do not receive gut decontamination per our institutional standard practice.
- Participants receiving GVHD prophylaxis with drugs other than calcineurin inhibitors, methotrexate or steroids, and agents listed above (e.g. abatacept).
- History of allergic reactions attributed to oral vancomycin or oral polymyxin B.
- Participants undergoing active therapy for immune-mediated or infectious colitis upon admission for allogeneic HSCT.
- Participants receiving antibiotic therapy for treatment of a bacterial infection or bacterial prophylaxis upon admission for allogeneic HSCT. Use of any agent (e.g. sulfamethoxazole/trimethoprim) for prophylaxis of *Pneumocystis jirovecii* pneumonia is permitted. Concurrent use of anti-fungal and anti-viral therapies is also permitted.

- Uncontrolled intercurrent illness including, but not limited to, ongoing or active infection or psychiatric illness/social situations that would limit compliance with study requirements.

Supplemental document 3 | CONSORT (*Con*solidated *S*tandards *o*f *R*eporting *T*rials) checklist

Section/Topic	Item No	Checklist item	Reported on page No.
**Title and abstract**			
	1a	Identification as a randomized trial in the title or abstract	1 (Title)<br>4 (abstract)
	1b	Structured summary of trial design, methods, results, and conclusions (for specific guidance see CONSORT for abstracts)	26-27, Figure 1
**Introduction**			
Background and objectives	2a	Scientific background and explanation of rationale	4, 7-9
	2b	Specific objectives or hypotheses	4, 26-27
**Methods**			
Trial design	3a	Description of trial design (such as parallel, factorial) including allocation ratio	26-27
	3b	Important changes to methods after trial commencement (such as eligibility criteria), with reasons	none
Participants	4a	Eligibility criteria for participants	26, 32, Supp. Document 2
	4b	Settings and locations where the data were collected	32
Interventions	5	The interventions for each group with sufficient details to allow replication, including how and when they were actually administered	26-27, Supp. Table 1
Outcomes	6a	Completely defined pre-specified primary and secondary outcome measures, including how and when they were assessed	26-27
	6b	Any changes to trial outcomes after the trial commenced, with reasons	none
Sample size	7a	How sample size was determined	26
	7b	When applicable, explanation of any interim analyses and stopping guidelines	N/A
Randomization:			
Sequence generation	8a	Method used to generate the random allocation sequence	26
	8b	Type of randomization; details of any restriction (such as blocking and block size)	26
Allocation concealment mechanism	9	Mechanism used to implement the random allocation sequence (such as sequentially numbered containers), describing any steps taken to conceal the sequence until interventions were assigned	N/A
Implementation	10	Who generated the random allocation sequence, who enrolled participants, and who assigned participants to interventions	26

Blinding	11a	If done, who was blinded after assignment to interventions (for example, participants, care providers, those assessing outcomes) and how	N/A
	11b	If relevant, description of the similarity of interventions	26
Statistical methods	12a	Statistical methods used to compare groups for primary and secondary outcomes	26, 31-32
	12b	Methods for additional analyses, such as subgroup analyses and adjusted analyses	26, 31-32
**Results**			
Participant flow (a diagram is strongly recommended)	13a	For each group, the numbers of participants who were randomly assigned, received intended treatment, and were analyzed for the primary outcome	Figure 1
	13b	For each group, losses and exclusions after randomization, together with reasons	Figure 1
Recruitment	14a	Dates defining the periods of recruitment and follow-up	26, 32
	14b	Why the trial ended or was stopped	N/A
Baseline data	15	A table showing baseline demographic and clinical characteristics for each group	Table 1
Numbers analyzed	16	For each group, number of participants (denominator) included in each analysis and whether the analysis was by original assigned groups	26, Fig 1
Outcomes and estimation	17a	For each primary and secondary outcome, results for each group, and the estimated effect size and its precision (such as 95% confidence interval)	Figures 3, 4 Table 2
	17b	For binary outcomes, presentation of both absolute and relative effect sizes is recommended	Table 2
Ancillary analyses	18	Results of any other analyses performed, including subgroup analyses and adjusted analyses, distinguishing pre-specified from exploratory	26-27
Harms	19	All important harms or unintended effects in each group (for specific guidance see CONSORT for harms)	13
**Discussion**			
Limitations	20	Trial limitations, addressing sources of potential bias, imprecision, and, if relevant, multiplicity of analyses	23-24
Generalizability	21	Generalizability (external validity, applicability) of the trial findings	24-25
Interpretation	22	Interpretation consistent with results, balancing benefits and harms, and considering other relevant evidence	20-25
**Other information**			
Registration	23	Registration number and name of trial registry	4, 32
Protocol	24	Where the full trial protocol can be accessed, if available	32
Funding	25	Sources of funding and other support (such as supply of drugs), role of funders	5, 34

Supplemental document 4 | Relevance of the vancomycin polymyxin B gut decontamination strategy to a larger study

For Gram negative coverage

Antibiotic prophylaxis with levofloxacin has been associated with a decrease in Gram-negative bacteremia in adult allo-HSCT recipients (7). In our study, all Gram-negative BSI's occurred in the no-GD arm. Furthermore, except for *Proteus, Serratia, and Burkholderia* spp. (8), polymyxin B and colistin (polymyxin E) are highly active against the family Enterobacteriaceae (which includes *Leclercia adecarboxylata* and *E. coli,* BSIs from patients C03 and C10, respectively) (3). This suggests that while the *Klebsiella* infection may not have been preventable due to colistin resistance, if GD had been effective at eliminating polymyxin B /colistin-sensitive organisms, then the *Leclercia adecarboxylata* and *E. coli* BSIs may have been prevented, with both strains having an MIC <1ug/mL.

For Gram-positive coverage by vancomycin:

The only GD subject to have a BSI was C11 which was sensitive to vancomycin. Our data shows that the BSI causing organism, *Lysinibacillus fusiformis*, was not found in any of the stool samples for this patient (C11) suggesting the BSI did not originate from the gut. In contrast to adult HSCT studies that show a predominance of Gram-negative bacteria in BSIs (7, 9), several studies in pediatrics demonstrate that Gram-positive organisms accounted for the largest percentage of BSI in the immunocompromised pediatric population (10-13). The largest single-center cohort of pediatric HSCT patients to examine the risk of BSI showed a predominance of Gram-positive bacteria (46% of the BSIs compared to 24% Gram-negative) (10). This is consistent with our study (6 Gram-positive BSI and 3 Gram-negative BSI) and a previous trial of 277 pediatric patients who received oral polymyxin B sulfate, where 75% of the BSI isolates were Gram-positive organisms (14). We have strong evidence that both the *MRSA* from patient C04 on day +5 and *E. faecium* from patient C22 on day +20 (of the 2nd transplant) may have come from the gut, and as noted above, the *S. epidermidis* may also have come from the gut as well based on temporal association. Furthermore, for those organisms that were Gram-positive BSIs, all were sensitive to vancomycin (Supplementary Table 6). This suggests that for the Gram-positive BSIs that likely originated from the gut, that these too may have been prevented with the vancomycin component of the GD strategy.

Supplemental References:

1. Humphries RM, Green DA, Schuetz AN, Bergman Y, Lewis S, Yee R, et al. Multicenter Evaluation of Colistin Broth Disk Elution and Colistin Agar Test: a Report from the Clinical and Laboratory Standards Institute. *Journal of Clinical Microbiology.* 2019;57(11):e01269-19.
2. CLSI. *Performance Standards for Antimicrobial Susceptibility Testing.*
3. (EUCAST) ECoAST. Colistin: clinical breakpoints, version 1.0. https://eucast.org/fileadmin/src/media/PDFs/EUCAST_files/Rationale_documents/Colistin_rationale_1.0.pdf. Accessed 9-July-2021.
4. Lander ES, and Waterman MS. Genomic mapping by fingerprinting random clones: A mathematical analysis. *Genomics.* 1988;2(3):231-9.
5. Alcock BP, Raphenya AR, Lau TTY, Tsang KK, Bouchard M, Edalatmand A, et al. CARD 2020: antibiotic resistome surveillance with the comprehensive antibiotic resistance database. *Nucleic Acids Res.* 2020;48(D1):D517-D25.
6. Alho AC, Kim HT, Chammas MJ, Reynolds CG, Matos TR, Forcade E, et al. Unbalanced recovery of regulatory and effector T cells after allogeneic stem cell transplantation contributes to chronic GVHD. *Blood.* 2016;127(5):646-57.
7. Stoma I, Littmann ER, Peled JU, Giralt S, van den Brink MRM, Pamer EG, et al. Compositional flux within the intestinal microbiota and risk for bloodstream infection with gram-negative bacteria. *Clinical Infectious Diseases.* 2020.
8. Olaitan AO, Morand S, and Rolain J-M. Mechanisms of polymyxin resistance: acquired and intrinsic resistance in bacteria. *Frontiers in Microbiology.* 2014;5(643).
9. Girmenia C, Bertaina A, Piciocchi A, Perruccio K, Algarotti A, Busca A, et al. Incidence, Risk Factors and Outcome of Pre-engraftment Gram-Negative Bacteremia After Allogeneic and Autologous Hematopoietic Stem Cell Transplantation: An Italian Prospective Multicenter Survey. *Clinical infectious diseases : an official publication of the Infectious Diseases Society of America.* 2017;65(11):1884-96.
10. Akinboyo IC, Young RR, Spees LP, Heston SM, Smith MJ, Chang Y-C, et al. Microbiology and Risk Factors for Hospital-Associated Bloodstream Infections Among Pediatric Hematopoietic Stem Cell Transplant Recipients. *Open Forum Infectious Diseases.* 2020;7(4).
11. Czyżewski K, Styczyński J, Giebel S, Frączkiewicz J, Salamonowicz M, Zając-Spychala O, et al. Age-dependent determinants of infectious complications profile in children and adults after hematopoietic cell transplantation: lesson from the nationwide study. *Ann Hematol.* 2019;98(9):2197-211.
12. Lake JG, Weiner LM, Milstone AM, Saiman L, Magill SS, and See I. Pathogen Distribution and Antimicrobial Resistance Among Pediatric Healthcare-Associated Infections Reported to the National Healthcare Safety Network, 2011-2014. *Infection control and hospital epidemiology.* 2018;39(1):1-11.
13. Kersun LS, Propert KJ, Lautenbach E, Bunin N, and Demichele A. Early bacteremia in pediatric hematopoietic stem cell transplant patients on oral antibiotic prophylaxis. *Pediatric blood & cancer.* 2005;45(2):162-9.
14. Sarashina T, Yoshida M, Iguchi A, Okubo H, Toriumi N, Suzuki D, et al. Risk factor analysis of bloodstream infection in pediatric patients after hematopoietic stem cell transplantation. *J Pediatr Hematol Oncol.* 2013;35(1):76-80.
